# Transcriptomic prey‐capture responses in convergently evolved carnivorous pitcher plants

**DOI:** 10.1111/nph.70848

**Published:** 2025-12-17

**Authors:** Takanori Wakatake, Kenji Fukushima

**Affiliations:** ^1^ Institute for Molecular Plant Physiology and Biophysics University of Würzburg Würzburg 97082 Germany; ^2^ Center for Frontier Research, National Institute of Genetics 1111 Yata Mishima Shizuoka 411‐8540 Japan; ^3^ Genetics Program Graduate Institute for Advanced Studies, SOKENDAI Mishima 411‐8540 Japan

**Keywords:** carnivorous plants, *Cephalotus follicularis*, convergent evolution, digestive protein, feeding response, molecular evolution, *Nepenthes gracilis*

## Abstract

- The Australian pitcher plant *Cephalotus* and the Asian pitcher plant *Nepenthes* exhibit striking morphological and functional similarities, serving as compelling examples of convergent evolution. Although trapping pitchers in both lineages represent some of the most elaborate leaf structures in angiosperms, it remains unknown whether their analogous phenotypes share common molecular foundations, especially at the level of gene expression.
- Here, we conducted tissue‐specific RNA‐seq experiments coupled with feeding treatments in *Cephalotus follicularis*, mirroring the available expression dataset of *Nepenthes gracilis* to analyze gene expression evolution underlying the phenotypic convergence.
- Functionally equivalent tissues in the two species tended to express similar gene sets, with common transcriptional responses that activate amino acid metabolism and protein synthesis upon the feeding treatment, yet with distinct transcriptional regulation of digestive enzyme genes. Additionally, we found multiple cases of combined convergence in expression and protein sequences in genes preferentially expressed in gland‐containing tissues.
- Our study showcases how common and unique transcriptional components are integrated to shape the independent emergence of complex leaf structures in angiosperms.

## Introduction

Convergent evolution, wherein distinct lineages independently evolve similar solutions to ecological pressures, offers insights into how organisms respond to natural selection. Among the most captivating examples are carnivorous plants, which collectively evolved in six plant orders of angiosperms (Freund *et al*., 2022). These remarkable organisms have evolved mechanisms for nutrient acquisition through the capture and digestion of prey, a strategy distinct from the soil‐based nutrient absorption typical of most land plants.

Most carnivorous plants have modified their leaves into trapping organs. To integrate trapping functions into leaves originally optimized for photosynthesis, considerable amounts of organ complexity have been added during carnivorous plant evolution, culminating in pitcher leaves, which represent one of the most intricate leaf structures observed in angiosperms (Tsukaya, 2014). Small arthropods that slip into this cavity become ensnared and are degraded by the cocktail of digestive enzymes secreted from glands. The released nutrients are absorbed from glands and then distributed throughout the plant body for growth and reproduction.

Caryophyllales encompasses a diverse and ancient lineage of carnivorous plants, yet pitfall traps evolved exclusively within *Nepenthes*. *Nepenthes* develops trapping pitchers in the distal half of a single leaf, which bears a tendrilled leaf‐blade‐like structure in the basal half. Thus, a single *Nepenthes* leaf is segmented along the proximodistal axis, featuring distinctive tissues each specialized for photosynthesis, carnivory, and its subfunctions, such as digestion. Gland cells on the inner wall of the digestive zone at the pitcher's base serve the dual purpose of secreting digestive enzymes and absorbing nutrients released during prey digestion (Freund *et al*., 2022). Above the digestive zone, the waxy zone hinders trapped prey from escaping, thanks to a slippery crystallized epicuticular wax (Gorb *et al*., 2005). The lid and the pitcher's rim, the peristome, secrete nectar to attract prey (Moran, 1996). The peristome also plays an important role in capturing prey by providing a slippery foothold for insects with wettable microscopic and macroscopic surface structures (Bohn & Federle, 2004; Labonte *et al*., 2021). Nutrients absorbed within the pitcher leaf are transported to the entire plant body through the tendril, which connects the pitcher to the leaf‐blade‐like tissue (flat part).

**Fig. 1:**
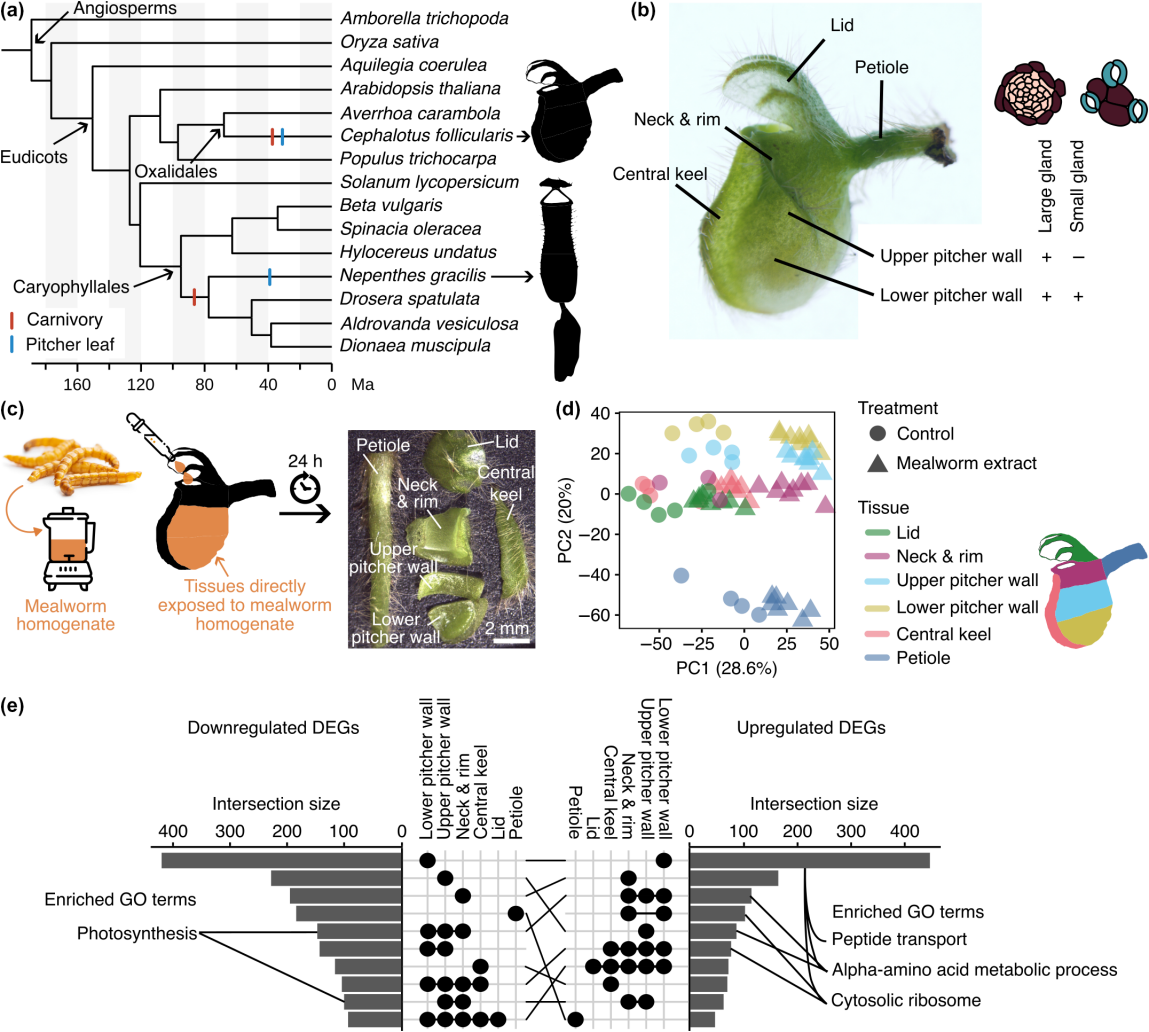
*Cephalotus* pitcher leaves respond to the feeding treatment. (a) The phylogenetic tree including 15 angiosperms used for analysis in this study. Color bars indicate the independent origins of plant carnivory and pitcher leaves in the Oxalidales and the Caryophyllales. The placement of the bars on the phylogenetic tree does not correspond to the estimated evolutionary timing of the trait's origin. (b) The tissue structure of the *Cephalotus* pitcher leaves. The positions of the two types of glands were indicated. (c) Illustration of the RNA‐seq experiment coupled with the mealworm extract treatment. (d) Principal component analysis of expression values of 3798 differentially expressed genes (DEGs) between control and worm extract‐treated samples. Points correspond to RNA‐seq samples. (e) The upset plot shows the number of upregulated (right panel) and downregulated (left panel) genes in each set association in the middle. Selected Gene Ontology (GO) terms enriched in the set association are indicated. The same set associations in the two upset plots were connected with black lines. Image credits: the mealworm photograph and icons from freepik.com. The photographs of the *Cephalotus* pitcher in (b) and (c) were reproduced from previous publications (Fukushima *et al*., 2021; Saul *et al*., 2023).

Despite evolving independently in distant lineages, *Cephalotus follicularis*, a carnivorous plant in the Oxalidales order and the exclusive member of the Cephalotaceae family, shares a remarkably similar structural and functional configuration of trapping pitchers with *Nepenthes* (Fig. 1a,b). *Cephalotus* develops carnivorous pitcher leaves alongside photosynthetic flat leaves depending on ambient temperature (Fukushima *et al*., 2017). The lower part of the pitcher leaves houses two distinct types of secretory glands (Freund *et al*., 2022). The small glands are densely clustered in glandular patches, which are situated symmetrically near the bottom of the pitcher leaves and can be distinguished by their colors and tissue thickness. The glandular patches are surrounded by a higher density of the large glands that are also distributed to the upper inner wall with gradually reduced density (Fig. 1b; Juniper *et al*., 1989). The downward‐pointing hairs on the neck prevent prey from escaping the cavity (Juniper *et al*., 1989). A roof‐like lid features nectary glands on its bottom side (Juniper *et al*., 1989; Vogel, 1998). With a few distinctions, such as gland morphology and distribution, *Cephalotus* pitchers share many hallmark characteristics with *Nepenthes* pitchers.

Carnivorous plants often exhibit a feeding response, which expedites prey digestion and nutrient absorption following prey capture. This response typically involves the reduction of pH in digestive fluids, enhancing the proteolytic activity of these fluids, the secretion of digestive enzymes, and the activation of nutrient absorption. Various cues can trigger this feeding response in distinct ways. For instance, in *Dionaea muscipula* (Venus flytrap) having snap trap, touch‐induced action potentials, repeated mechanical stimuli from struggling trapped prey, and chemicals released from degraded prey trigger trap movement, digestive enzyme secretions, and nutrient absorption (Libiaková *et al*., 2014; Bemm *et al*., 2016; Böhm *et al*., 2016; Pavlovič *et al*., 2017). In contrast to *Dionaea*, pitcher leaves do not rely on mechanical stimuli and use chemical cues to detect the presence of prey (Gallie & Chang, 1997). Chitin, a primary component of insect exoskeletons, as well as ammonium released during protein digestion and proteins, such as bovine serum albumin (BSA), each induce distinctive feeding responses in *Nepenthes* (Yilamujiang *et al*., 2016; Saganová *et al*., 2018). BSA and ammonium strongly induce digestive activities also in *Sarracenia purpurea*, a carnivorous pitcher plant in Ericales (Gallie & Chang, 1997).

In this study, we generated a tissue‐specific RNA‐seq dataset for *Cephalotus* under feeding treatment conditions and paired it with a mirrored dataset from *Nepenthes* (Saul *et al*., 2023). This approach enabled a direct comparison of transcriptomic features between these two deeply diverged, independently evolved pitcher plant lineages. Our analyses uncovered shared expression profiles as well as contrasting regulatory responses associated with prey capture. In addition, we identified convergent amino acid substitutions in 11 proteins upregulated or specifically expressed in gland‐containing tissues, suggesting potential adaptive evolution at both expression and protein sequence levels. We discuss the evolutionary implications of these shared and divergent molecular features that underpin the complex pitcher organizations.

## Materials and Methods

### Plant materials and growth conditions

Plants of *Cephalotus follicularis* Labill. were vegetatively propagated from the strain whose genome was sequenced previously (Fukushima *et al*., 2017) and grown in the ½‐strength Murashige & Skoog medium (½MS) supplemented with 3% sucrose, 1× Gamborg's vitamins, 0.1% 2‐(N‐morpholino)ethanesulfonic acid, 0.05% Plant Preservative Mixture (Plant Cell Technology, Washington, DC, USA), and 0.3% Phytagel at 25°C under long‐day conditions (16 h : 8 h, light : dark).

### Tissue‐specific RNA‐seq with the mealworm extract treatment

One hundred milligrams of dried mealworms (batch no.: L400518; MultiFit Tiernahrungs GmbH, Krefeld, Germany) were powdered with mortars and pestles. The dried mealworms were not sterile, although the drying process was expected to eliminate most viable microbes. The mealworm powder was dissolved in 1 ml of MilliQ water and vigorously vortexed. After the brief centrifugation, the supernatant was used for sample treatment. Approximately 10–30 μl of the mealworm extracts or Milli Q water (control) was supplied to open pitchers depending on the size of the pitcher. After 24 h, pitchers were sampled and dissected as described in Fig. 1(c) using small blades, then snap‐frozen in liquid nitrogen, and stored at −80°C. Dissecting one pitcher took *c*. 90 s. The number of pitchers per replicate was 30–36, with four biological replicates for the control and eight biological replicates for the treatment. Total RNA was extracted using PureLink Plant RNA Reagent (Thermo Fisher Scientific, Waltham, MA, USA) according to the manufacturer's instructions, and then further cleaned using the RNeasy Mini Column (Qiagen). During cleanup, on‐column DNA digestion was also performed using DNase I according to the manufacturer's instructions (Qiagen). Library preparation and sequencing (paired‐end, 150 bp) on NovaSeq 6000 (Illumina) were performed by Novogene.

### Differential gene expression analysis and gene ontology enrichment analysis

RNA‐seq reads were quality‐filtered using fastp v.0.23.2 (Chen *et al*., 2018) with default parameters and pseudo‐aligned to the *C. follicularis* transcriptome (Fukushima *et al*., 2017) using kallisto v.0.48.0 (Bray *et al*., 2016) with default settings. Cross‐species trimmed mean of *M*‐values (TMM) normalization of the count was performed using Amalgkit v.0.12.3 (https://github.com/kfuku52/amalgkit; Fukushima & Pollock, 2020). The obtained Fragments Per Kilobase of transcript per Million mapped reads (FPKM) values were used to plot heatmaps. Differentially expressed genes (DEGs) were detected by comparing control and mealworm extract‐treated samples for each tissue using the tcc package v.1.36.0 (Sun *et al*., 2013), which internally utilizes edgeR v.4.0.16 and TMM‐normalization (Robinson *et al*., 2010). Low‐expression genes were excluded using the filterLowCountGenes function before TMM‐normalization. Read count data for *Nepenthes gracilis* Korth. were obtained from the previous publication (Saul *et al*., 2023), and DEG analysis was performed using the same procedure as for *C. follicularis*. Gene Ontology (GO) enrichment analysis was conducted with clusterProfiler v.4.10.0 (Wu *et al*., 2021). GO terms for *C. follicularis* genes were assigned using EnTap v.0.10.8 (Hart *et al*., 2020), with homology searches performed against the TrEMBL, SwissProt (https://www.uniprot.org/), and NCBI plant RefSeq (https://ncbi.nlm.nih.gov/refseq/) databases. Annotations were selected based on Viridiplantae or Eukaryotes classifications in EggNOG.Tax.Scope. GO term information for *Nepenthes gracilis* was sourced from the previous publication (Saul *et al*., 2023).

### Self‐organizing map clustering

Self‐organizing map (SOM) clustering was performed with the kohonen package v.3.0.12 (Wehrens & Buydens, 2007). Genes with the top 10% coefficient variation in TMM‐normalized FPKM values (> 1 at least in one condition) were used for SOM clustering. FPKM values were mean‐centered and variance‐scaled, then used for SOM clustering in a 3 × 4 grid in hexagonal topology.

### Species tree inference

First, we inferred a phylogenetic tree of 15 species, including *C. follicularis* and Caryophyllales carnivorous plants (Fig. 1a; Supporting Information Table S1), following the essentially same procedures used in the previous publication (Saul *et al*., 2023). Briefly, common single‐copy BUSCO genes (v.5.3.2; embryophyta_odb10; Manni *et al*., 2021) among 15 species were aligned in‐frame with mafft v.7.508 (Katoh & Standley, 2013) and tranalign from Emboss v.6.6.0.0 (Rice *et al*., 2000). After trimming with trimAl v.1.4.rev15 (Capella‐Gutiérrez *et al*., 2009), all gene alignments were concatenated and used for maximum likelihood tree reconstruction under the GTR + R4 model with Iq‐Tree2 v.2.2.0.3 (Nguyen *et al*., 2015). The obtained tree was rooted using *Amborella trichopoda* as an outgroup and had the same topology as the previous publication (Saul *et al*., 2023). Next, divergence time estimation was performed with MCMCtree from the paml package v.4.9 (Yang, 2007) using the same parameters as the previous publication (Saul *et al*., 2023). The reconstructed species tree and concatenated codon alignment were used as inputs. Branch lengths and substitution model parameters were initially estimated using BASEML with a global clock and the GTR + G model.

### Orthogroup classification

**Fig. 2:**
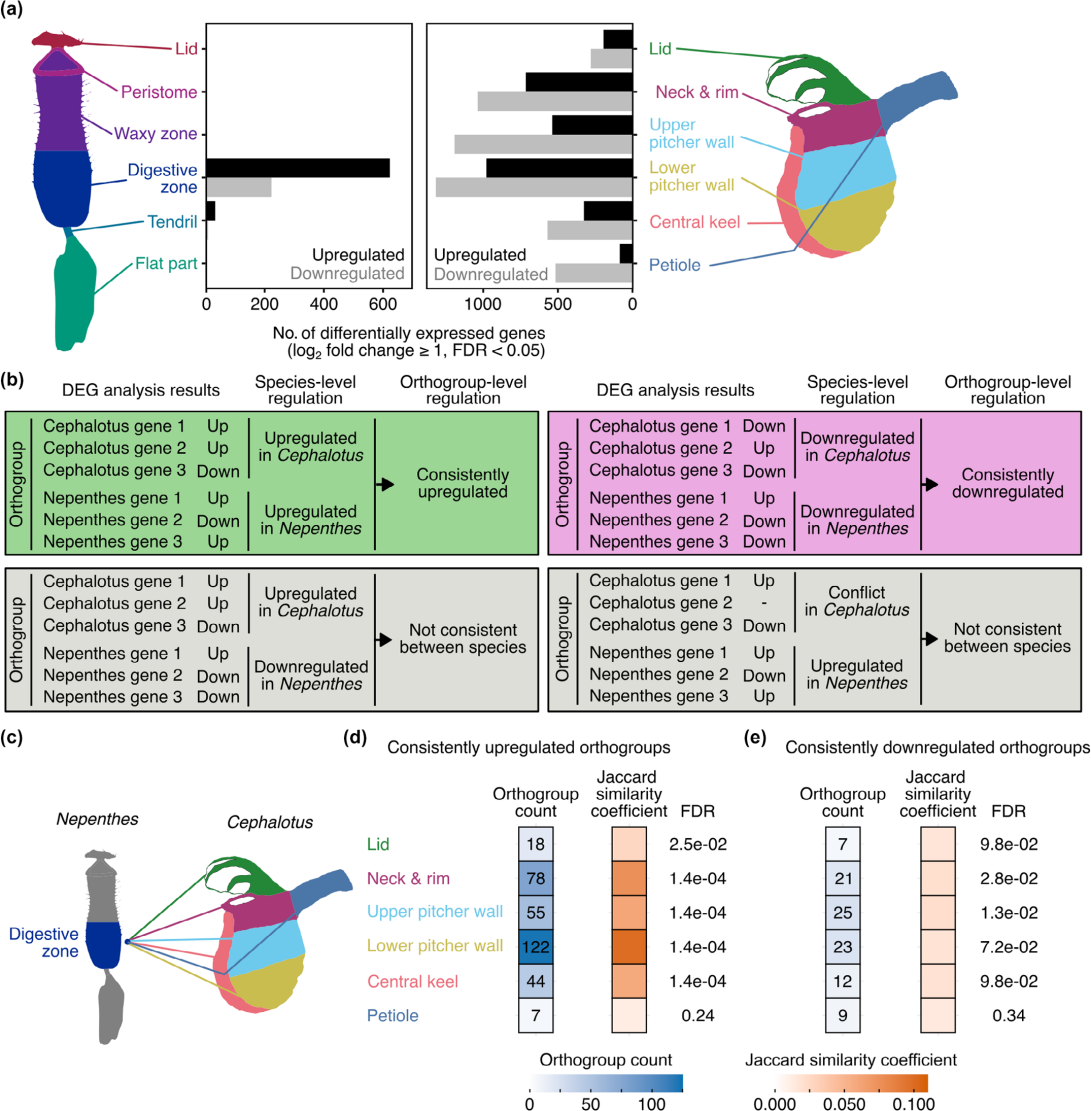
*Cephalotus* pitchers upregulate the gene set similar to *Nepenthes* pitchers in response to the feeding treatment. (a) The number of differentially expressed genes (DEGs) in each tissue in *Nepenthes gracilis* (left) and *Cephalotus follicularis* (right). Different tissues are indicated with different colors in the illustration. (b) The definition of the consistently upregulated (green) and downregulated (magenta) orthogroups in two species. Two examples with inconsistent regulation (gray) are shown in the second row. (c) Schematic comparison of DEGs between the digestive zone of *N. gracilis* and all pitcher tissues of *C. follicularis*. (d, e) Consistently upregulated and downregulated orthogroups were analyzed, respectively. The heatmaps indicating the number of shared orthogroups and the Jaccard similarity coefficient (JC, middle), and false discovery rate (FDR) values for JC (right) were shown. The numbers in the tiles indicate the shared orthogroup count between two groups. FDR values were calculated using the Benjamini–Hochberg method for multiple testing correction, following *P* value computation via permutation tests.

**Fig. 3:**
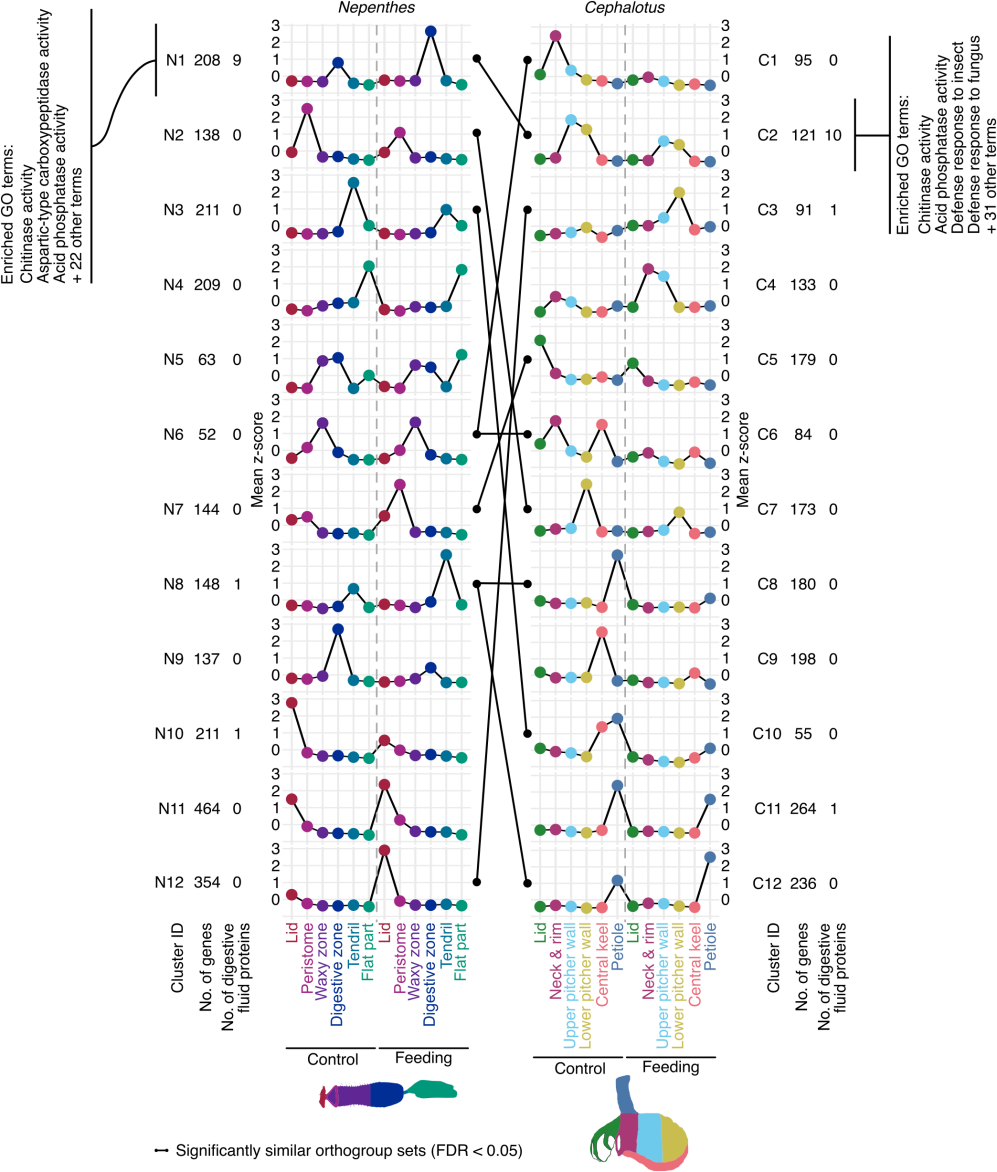
Comparison of tissue‐specific gene expression in *Nepenthes gracilis* and *Cephalotus follicularis* pitchers. Self‐organizing map (SOM) clustering was performed on genes with high coefficients of variation in expression across six pitcher tissues in *N. gracilis* (left) and *C. follicularis* (right). The *y*‐axis represents the average *z*‐score of gene expression within each cluster. Vertical dotted lines indicate the boundary between control and feeding conditions. Cluster IDs are prefixed with N for *Nepenthes* and C for *Cephalotus*. Clusters with significantly similar orthogroup compositions between the two species (Jaccard similarity coefficient, false discovery rate (FDR) < 0.05) are connected by black lines. Selected enriched Gene Ontology (GO) terms for representative clusters are also shown. FDR values were calculated using the Benjamini–Hochberg method for multiple testing correction after *P* values were obtained through permutation tests.

Orthogroup classification was performed with OrthoFinder v.2.5.4 (Emms & Kelly, 2019) using the inferred species tree as a guide. We selected orthogroups that included genes from at least 30% of the species in our dataset, resulting in a total of 13 023 orthogroups for downstream analyses. The hierarchical orthogroup classification at the node representing the most recent common ancestor of *C. follicularis* and *N. gracilis* was used to assess shared DEGs and SOM cluster members between the two species (Figs 2, 3, S1–S3).

### Gene tree inference

For each orthogroup, a gene phylogenetic tree was inferred with Iq‐Tree2 v.2.2.0.3 under the GTR + G4 model using in‐frame aligned and trimmed CDS sequences with mafft v.7.508 (Katoh & Standley, 2013), tranalign from Emboss v.6.6.0.0 (Rice *et al*., 2000), and ClipKit v.1.3.0 (Steenwyk *et al*., 2020). Stop codons and ambiguous codons were replaced with gaps, and codon sites with many gaps were removed using ‘mask’ and ‘hammer’ functions in Cdskit v.0.10.2, respectively (https://github.com/kfuku52/cdskit). Obtained gene phylogenetic trees were reconciled with the inferred species tree using GeneRax v.2.0.4 (−‐rec‐model ‘UndatedDL’; Morel *et al*., 2020). Divergence times of individual species‐tree‐aware gene phylogenetic trees were estimated with Radte v.0.2.0 (https://github.com/kfuku52/RADTE) using the dated species tree as a reference (Fukushima & Pollock, 2020). Branching events in the gene trees were classified as either speciation or duplication using the species‐overlap method (Huerta‐Cepas *et al*., 2007).

### Jaccard similarity coefficient calculation

Jaccard similarity coefficient was calculated by dividing the size of the intersection by the size of the union of two hierarchical orthogroup sets. *P* values were calculated as the probabilities where 10 000 sets of simulated values exceeded observed values. Simulations were performed by permuting hierarchical orthogroups used to calculate the original Jaccard similarity coefficient. To ensure consistency, contradictory hierarchical orthogroup assignments (e.g. assigning the same hierarchical orthogroup to both upregulated and downregulated categories within the same tissue) were avoided during the simulations. Multiple testing corrections were performed using the p.adjust function with the Benjamini–Hochberg method in R v.4.3.2.

### Convergent amino acid substitution detection

We selected orthogroups that include both *N. gracilis* and *C. follicularis* genes upregulated in the digestive zone or the lower pitcher wall (false discovery rate (FDR) < 0.05, log_2_ fold change > 1), or specifically expressed in the gland‐containing tissues (SOM clusters N1 and N9 in Fig. 3 for *N. gracilis* genes, and SOM clusters C2, C3, and C7 in Fig. 3 for *C. follicularis* genes). Although the hierarchical orthogroup, which further splits the nonhierarchical orthogroup at each hierarchical level based on the species tree, is more accurate, it may separate distantly related branch pairs into two different groups, excluding these pairs from the analyses. We used nonhierarchical orthogroups to encompass such branch pairs within the same orthogroup. By using nonhierarchical orthogroups, we detected excess convergence in key proteins, such as HAK5, where the convergent branch pair split into two separate hierarchical orthogroups. Csubst v.1.1.4 estimated protein convergence for each selected orthogroup based on a codon alignment, a dated and reconciled gene phylogenetic tree, and a reconstructed ancestral codon state (Fukushima & Pollock, 2023). Ancestral codon reconstructions were performed with Iq‐Tree2 v.2.2.0.3 using the ‘‐‐ancestral’ option (Nguyen *et al*., 2015). Although several digestive enzymes have been reported in *Drosera adelae* F.Muell., the absence of a genome assembly led us to exclude this species from the initial orthogroup classification and the subsequent CSUBST analysis.

### Homologous gene search

Homologous genes were searched by running Tblastx v.2.13.0 with an *E*‐value threshold of 0.01 against 15 species CDS sequences used in the species tree inference. This additional search was performed for specific gene family analyses, such as those of digestive fluid proteins, where homologs needed to be identified beyond the orthogroups defined by OrthoFinder. Gene phylogenetic trees were inferred through the same procedure used for the orthogroup analysis, as described previously.

## Results and Discussion

### Prey‐induced transcriptional responses of *Cephalotus* pitcher tissues

To explore how *Cephalotus* responds to prey capture at the transcriptome level, we performed an RNA‐seq experiment with a feeding treatment. To mimic prey capture, we supplied pitchers with the water extraction of dried mealworms. As a negative control, water was supplied. After 24 h, the treated pitchers were dissected into six parts (lid, neck and rim, upper pitcher wall, lower pitcher wall, central keel, and petiole) to examine tissue‐specific responses, following the dissection scheme established in the previous publication (Saul *et al*., 2023), and the dissected tissues were subjected to the RNA‐seq experiment (Fig. 1c). To overview the dataset, a principal component analysis (PCA) was performed using DEGs that responded to the feeding treatment in at least one tissue (3798 genes, FDR < 0.05, |log_2_ fold change| ≥ 1.0; Table S2). The first Principal Component 1 (PC1) explained 28.6% of the variation, clearly separating control and fed samples in all tissues (Fig. 1d). The pitcher tissues were separated mainly with the PC2 axis. The number of unique DEGs (i.e. genes differentially expressed only in the focal tissue) was highest in the lower pitcher wall (Fig. 1e), where two types of glands are located (Fig. 1b). In this tissue, GO terms ‘peptide transport’, ‘alpha‐amino acid metabolic process’, and ‘cytosol ribosome’ were significantly enriched in the upregulated DEGs, suggesting a role in absorbing and assimilating proteinaceous degradation products and protein synthesis (Fig. 1e; Table S3). The term ‘alpha‐amino acid metabolic process’ was also enriched in the upregulated DEGs in the upper pitcher wall, in correlation with the location of the large glands. ‘Cytosol ribosome’ showed enrichment in all analyzed tissues but the petiole (Fig. 1e), suggesting that protein synthesis is activated in the whole pitcher in response to the feeding treatment but is decoupled in the petiole. Among the commonly downregulated genes, we found that the GO term ‘photosynthesis’ was enriched in the pitcher‐forming tissues (Fig. 1e). While prey capture generally enhances photosynthesis in carnivorous plants over the long term (2 wk to 5 months) by increasing nutrient availability (Givnish *et al*., 1984; Farnsworth & Ellison, 2008; Pavlovič *et al*., 2009, 2011; He & Zain, 2012), our results indicate a more immediate transcriptional response that prioritizes carnivory over photosynthesis. This response appears mechanistically distinct from that observed in *Di. muscipula*, where photosynthetic rates rapidly decline upon trigger hair stimulation but recover within 10 min (Pavlovič *et al*., 2010). The gene expression changes in *Cephalotus* suggest a more sustained downregulation, potentially reflecting a resource allocation strategy that enhances digestive processes.

### Feeding responses in the two pitcher plant lineages

We previously conducted tissue‐specific RNA‐seq experiments with the same feeding treatment in the pitcher leaves of *N. gracilis*, a carnivorous pitcher plant that evolved separately from *Cephalotus* (Fig. 1a; Saul *et al*., 2023), providing an opportunity to test whether independently evolved carnivorous pitcher leaves exhibit similar transcriptome responses upon prey capture. To enable direct comparison, we reanalyzed the *N. gracilis* dataset using the same method as for *Cephalotus*, and used the resulting output for downstream analyses (Table S4). In contrast to *N. gracilis* pitcher, in which DEGs were detected almost exclusively in the digestive zone, *Cephalotus* showed DEGs in all analyzed pitcher tissues (Fig. 2a). The number of DEGs was the highest in the lower pitcher wall, which, together with the upper pitcher wall, was directly exposed to the mealworm extract. DEG abundance decreased progressively with the increasing distance from the treated site and reached its lowest in the most distant tissues, the petiole and the lid, although it never dropped entirely to zero (Fig. 2a). To integrate the gene expression between the two pitcher plant species split more than 100 million years ago (Saul *et al*., 2023), gene orthology was inferred using OrthoFinder with the inferred species tree (Fig. 1a) to inform phylogenetic relationships (Table S5; Emms & Kelly, 2019). We defined the orthogroup‐level feeding response as either up, down, or conflict by majority decision of member DEGs within orthogroups for each pitcher tissue (Fig. 2b) to evaluate the overlapped responses between the digestive zone of *N. gracilis* and all pitcher tissues of *C. follicularis* (Fig. 2c). The lower pitcher wall showed the largest number of orthogroups consistently upregulated in the digestive zone in *N. gracilis*, exhibiting the highest Jaccard similarity coefficient, which measures the degree of overlap between two orthogroup sets (Fig. 2d,e). A large portion of enriched GO terms in these shared orthogroups was related to the ribosome (Table S6). These analyses suggested a shared response to the feeding treatment in two phylogenetically distant pitcher plants.

### Tissue‐specific gene expression in the two pitcher plant lineages

Distinct tissues contribute to the various subfunctions that underpin the highly organized pitcher leaves. To compare tissue‐specific gene expression and feeding responses in the two pitcher plant lineages, we applied SOM clustering to the merged RNA‐seq datasets from both control and feeding conditions. Genes with a high coefficient of variation in gene expression (top 10%, 1809 genes for *C. follicularis*, 2339 genes for *N. gracilis*) were subjected to SOM clustering in a 4 × 3 grid with hexagonal topology (Table S7).

In *N. gracilis*, cluster N1 exhibited high and specific expression in the digestive zone and included nine out of the 18 genes orthologous to those encoding digestive fluid proteins in congeneric species (*N. alata*, *N. rafflesiana*, *and N. mirabilis*; Fig. 3; Saul *et al*., 2023). Similarly, in *C. follicularis*, cluster C2 exhibited high expression specifically in the upper and lower pitcher walls and included 10 out of 12 genes encoding previously identified digestive fluid proteins (Fig. 3). Both clusters were enriched in GO terms associated with digestive functions, such as ‘chitinase activity’, ‘aspartic‐type endopeptidase activity’, and ‘acid phosphatase activity’, suggesting a functional link to the digestive processes (Table S8). Cluster C2 of *C. follicularis* additionally exhibited enrichment for defense‐related GO terms (e.g. ‘defense response to insect’ and ‘defense response to fungi’), likely reflecting an overlap between digestive physiology and immune functions. Other clusters also displayed tissue‐biased expression patterns. These results demonstrated that SOM clustering effectively groups genes into biologically meaningful modules potentially associated with distinct tissue roles and feeding responses.

To quantitatively assess the similarity of tissue‐specific gene expressions between the two species, we calculated the Jaccard similarity coefficient for orthogroup sets in a pair of SOM clusters from *N. gracilis* and *C. follicularis* (Figs 3, S1). As expected from shared expression of genes encoding digestive fluid proteins, *N. gracilis* cluster N1, which exhibited high expression in the digestive zone, showed significant similarity to *C. follicularis* cluster C2. *Nepenthes* clusters N3 and N8, which showed high expression in the tendril, were most similar to *C. follicularis* clusters C10, C8, and C12, respectively, which were highly expressed in the petiole. Both tissues connect the pitcher to the main plant body. Similarly, *N. gracilis* cluster N6, which showed peak expression in the waxy zone, displayed notable similarity to *C. follicularis* clusters C1 and C6, where expression peaked in the neck and rim tissues (Fig. 3). These tissues develop extracellular structures that prevent prey from escaping (Juniper *et al*., 1989; Gorb *et al*., 2005). Together, these results indicate that functionally equivalent pitcher tissues tend to express similar gene sets.

### Feeding responses of genes encoding digestive fluid proteins

**Fig. 4:**
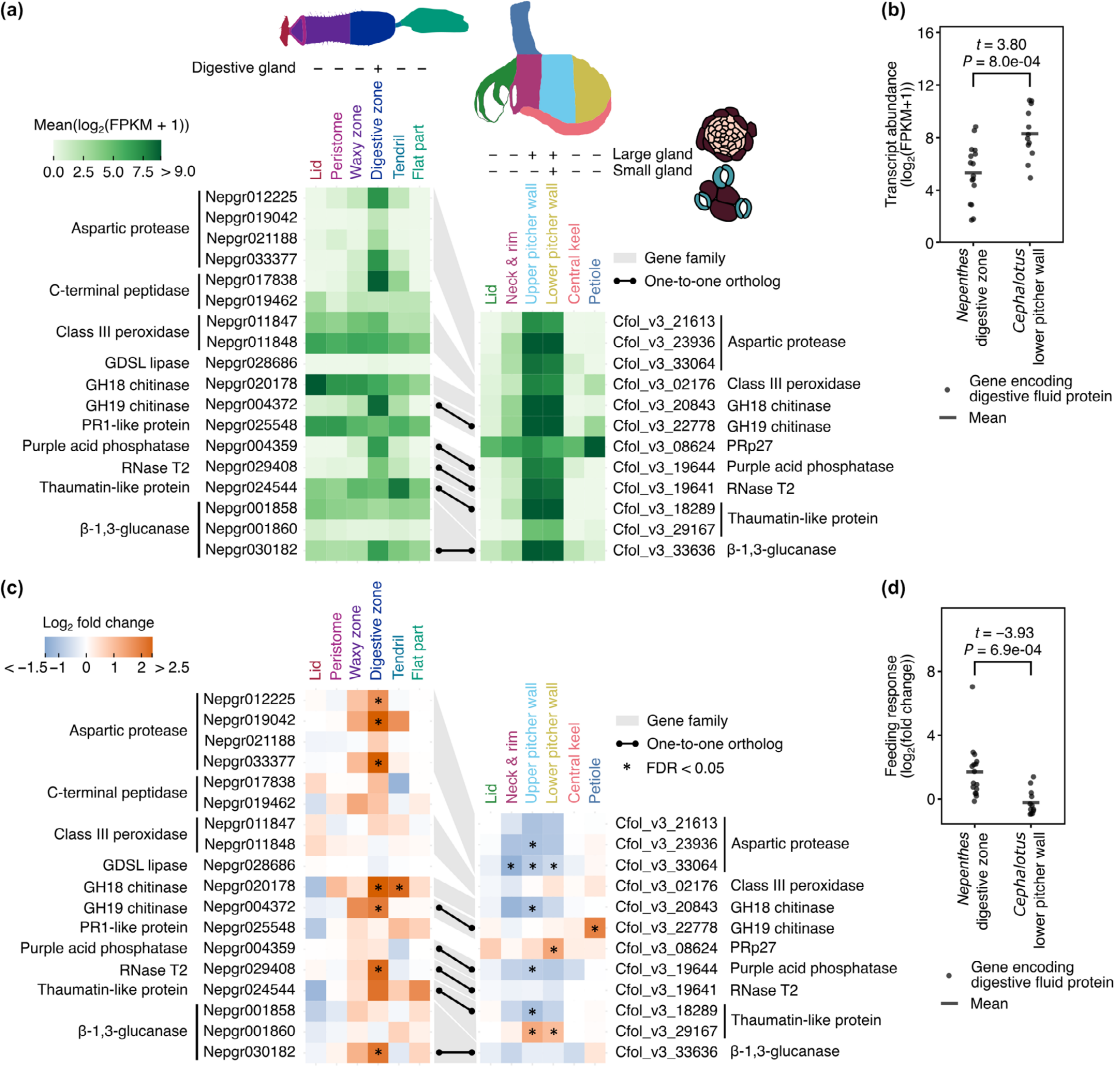
Differential response of genes encoding digestive fluid proteins to the feeding treatment in *Cephalotus* and *Nepenthes* pitchers. (a) Tissue‐specific transcript abundance (log_2_(FPKM + 1)) of genes encoding digestive fluid proteins. (b) Expression levels of the genes encoding digestive fluid proteins in the digestive zone and the lower pitcher wall before the feeding treatment. (c) Tissue‐specific log_2_ fold change of genes encoding digestive fluid proteins upon the feeding treatment. (d) Log_2_ fold change of the genes encoding digestive fluid proteins in the digestive zone and the lower pitcher wall. Gray ribbons connect genes in the same gene family. The black dots and lines connect the orthologous genes. Asterisks in (c) indicate false discovery rate (FDR) < 0.05 in differential gene expression analysis. *P* values and *t* statistics of Welch's two‐sided *t*‐tests are shown above the plot in (b) and (d).

One of the well‐documented feeding responses in various carnivorous plants is the upregulation of secreted proteins in the digestive cocktail. We previously reported tissue‐specific expression patterns of genes encoding digestive fluid proteins in *C. follicularis* under the control condition (Saul *et al*., 2023); however, their responses to prey capture remain unexplored. We examined the transcriptional responses of these genes and compared them with *N. gracilis* genes encoding proteins orthologous to experimentally confirmed digestive fluid proteins in other *Nepenthes* species. In *C. follicularis*, all examined genes, except for *PRp27*, which encodes an immune‐responsive metalloprotein (Morimoto *et al*., 2022), showed high tissue‐specific basal expression in both the upper and lower pitcher walls, mirroring the distribution of the large gland (Fig. 4a). In *N. gracilis*, approximately half of the orthologs encoding digestive fluid proteins exhibited highly specific expression in the digestive zone even before feeding treatment (Fig. 4a). These expression patterns suggest constitutive protein production, consistent with the prevalence of digestive fluid proteins in unfed pitchers (Buch *et al*., 2015; Saganová *et al*., 2018; Dkhar *et al*., 2020). Basal expression levels of these genes were significantly higher in *C. follicularis* than in *N. gracilis* (Fig. 4b).

Next, we compared the feeding responses of the genes encoding digestive fluid proteins. Low‐expression genes (log_2_(FPKM + 1) < 1.0) in gland‐containing tissues both before and after the feeding treatment were excluded from these analyses. Surprisingly, in *C. follicularis*, these genes tended to be downregulated after the feeding treatment (2/5/5, upregulated/nonsignificant/downregulated, FDR < 0.05) (Fig. 4c,d). By contrast, in *N. gracilis*, seven digestive fluid protein orthologs were upregulated specifically in the digestive zone following feeding treatment (7/11/0, upregulated/nonsignificant/downregulated, FDR < 0.05). These expression profiles underscore the distinct transcriptional regulations between the two pitcher plant lineages; in *N. gracilis*, a majority of genes encoding digestive fluid proteins are induced by feeding, whereas in *Cephalotus*, they tend to exhibit constitutive expression before prey capture (Fig. 4b) and are downregulated after feeding (Fig. 4d).

Interestingly, in *C. follicularis*, genes encoding a precursor of immune‐eliciting peptide and elicitor‐producing apoplastic proteases showed expression profiles similar to genes encoding digestive fluid proteins, suggesting continuous production of immune elicitors (Notes S1 and Figs S2–S4). Also, while jasmonic acid (JA) signaling plays a central role in regulating digestive enzyme secretion in carnivorous Caryophyllales, our transcriptomic analysis suggests a more limited or divergent involvement of JA in *C. follicularis* (Notes S2; Fig. S5). Together, these examples highlight lineage‐specific regulatory mechanisms underlying carnivorous responses, despite their common reliance on defense‐related enzymes.

The downregulation of the *Cephalotus* genes encoding digestive fluid proteins contrasts with the common strategy observed in many other carnivorous plants, where digestion is transcriptionally enhanced following prey capture. In *Nepenthes*, the aspartic protease nepenthesin is transcriptionally upregulated after feeding, leading to higher protein abundance and proteolytic activity in the digestive fluid (Saganová *et al*., 2018). Notably, both total proteolytic activity and aspartic protease abundance of Cfol_v3_21613 and Cfol_v3_23936, as individually assessed by immunodetection, in the digestive fluid remain nearly constant for 7 d postfeeding in *C. follicularis* (Pavlovič *et al*., 2024), indicating that transcriptional repression does not immediately impact the proteolytic capacity of the digestive fluid. The high basal expressions before feeding may buffer the digestive capacity in the early stages of prey breakdown. The observed downregulation may reflect a resource reallocation from digestion to nutrient assimilation following prey acquisition. Consistent with this idea, genes associated with nitrogen assimilation were upregulated after the feeding treatment (Notes S3; Fig. S6). However, as our analysis was limited to a single time point, it remains unclear whether this downregulation is sustained or transient. Future time‐course experiments will be necessary to clarify the temporal dynamics of digestive fluid protein expression.

### Convergent protein evolution in genes expressed in gland‐containing tissues

While gene expression evolution is one aspect of adaptive evolution, changes in protein sequences represent another, reflecting potential adaptations at the biochemical level. Gene expression in specialized tissues, such as the gland‐bearing structures of pitcher plants, may cause proteins to adapt to new cellular contexts, potentially leading to the coevolution of gene expression patterns and protein sequences. Indeed, *C. follicularis* and carnivorous Caryophyllales (including *Nepenthes*) exhibit convergent amino acid substitutions in digestive enzymes (RNase T2, GH19 chitinase, and purple acid phosphatase) that likely alter their biochemical properties for prey digestion (Nishimura *et al*., 2013; Fukushima *et al*., 2017; Fukushima & Pollock, 2023). To investigate these combined molecular signatures of convergent evolution in pitcher plants, we searched for convergent amino acid substitutions among genes expressed in gland‐containing tissues in *C. follicularis* and *N. gracilis* using CSUBST (Fukushima & Pollock, 2023). CSUBST calculates the *ω*_C_ metric, which estimates the rate of protein convergence. With the neutral expectation of 1.0, higher *ω*_C_ values indicate accelerated rates of protein convergence. For this analysis, we selected 173 orthogroups containing genes upregulated in the digestive zone or in the lower pitcher wall (FDR < 0.05, log_2_ fold change > 1), or specifically expressed in tissues with glands (SOM clusters N1 and N9 for *N. gracilis* or SOM clusters C2, C3, and C7 for *C. follicularis*). With the thresholds for the number of convergent amino acid substitutions (*O*_C_^N^ > 3.0) and for protein convergence rates (*ω*_C_ > 3.0) to the branch pairs leading to *C. follicularis* and *N. gracilis* genes, we obtained 11 convergent branch pairs from 11 orthogroups (Table S9). Although a different species set was used compared with the previous study (Fukushima & Pollock, 2023), we successfully obtained high *ω*_C_ values in RNase T2 (OG0001136) in *C. follicularis* and carnivorous Caryophyllales. In addition, the result included two proteins found in digestive fluids, S1‐P1 nuclease and polygalacturonase‐inhibiting protein (PGIP; Schulze *et al*., 2012; Lee *et al*., 2016; Kocáb *et al*., 2020; Arai *et al*., 2021), and HIGH AFFINITY K^+^ TRANSPORTER 5 (HAK5) from the KT/HAK/KUP family, which functions in potassium uptake in the glands of *Di. muscipula* (Scherzer *et al*., 2015).

**Fig. 5:**
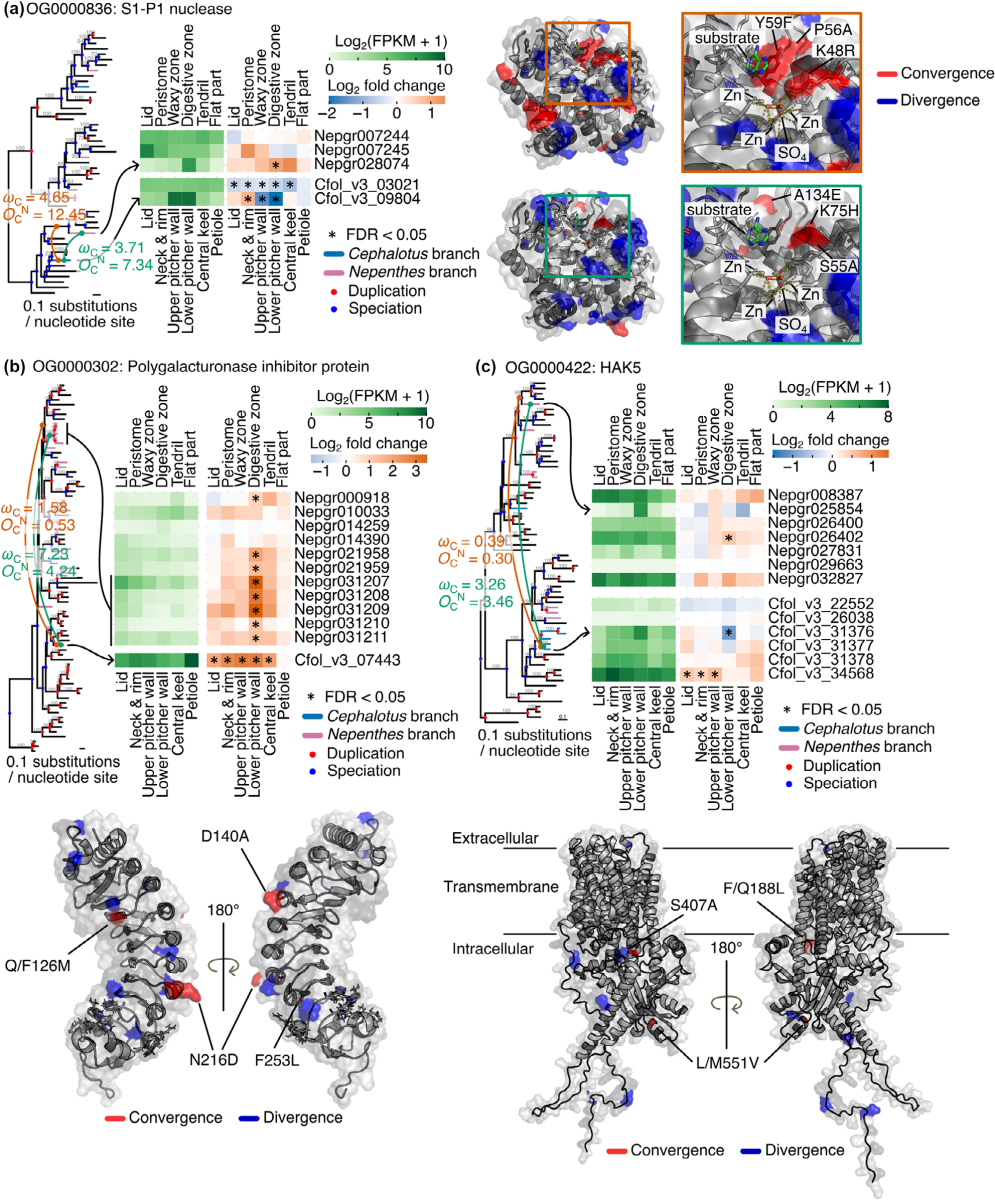
Convergent amino acid substitutions in proteins expressed in the gland‐containing tissues. S1‐P1 nuclease (a), Polygalacturonase‐inhibiting protein (PGIP) (b), and K^+^ transporter HAK5 (c) are shown. *Cephalotus* and *Nepenthes* lineages are highlighted in blue and purple, respectively. Green lines connect focal branch pairs that exhibit high convergence rates, while brown lines link the focal *Cephalotus* branches to the stem branches of carnivorous Caryophyllales orthologs. The corresponding *ω*_C_ and *O*_C_^N^ (protein convergence rate and the number of amino acid‐changing convergent substitutions, respectively) values are indicated in matching colors. Ultrafast bootstrap values from IQ‐TREE are indicated above the branches. Branches reconciled by GeneRax are marked with a hyphen (–). Node colors represent inferred evolutionary events: speciation (blue) and gene duplication (red). Expression levels (log_2_(FPKM +1)) under the basal condition and log₂ fold changes upon the feeding treatment are shown as heatmaps. Asterisks denote significant differential expression (false discovery rate (FDR) < 0.05). Convergent and divergent amino acid substitutions are mapped onto the protein structures and highlighted in red and blue, respectively. The protein structures were obtained from the Protein Data Bank entry or the AlphaFold Protein Structure Database (a, 3W52; b, 6W78; c, AF‐Q5JK32‐F1‐v4). Site numbers correspond to positions in the protein structures. Note that the predicted HAK5 protein structure has very low pLDDT scores (< 50) in the cytoplasmic N‐terminal (residues 1–55) and C‐terminal regions (residues 670–706), indicating lower confidence in those areas. Black lines are drawn to indicate extracellular, transmembrane, and intracellular regions, but these are intended only as rough guidelines and do not reflect precise structural boundaries. For S1‐P1 nuclease (a), two separate mapping patterns are displayed: one for the focal *Cephalotus*–*Nepenthes* branch pair, and another for the *Cephalotus* branch paired with the stem branch of carnivorous Caryophyllales ortholog. Magnified views of the substrate‐binding sites are provided, with frame colors corresponding to the line colors linking branches in the tree. Substrate adenine is shown as green sticks. Only convergent substitutions near the substrate‐binding site are labeled.

S1‐P1 nucleases have been detected in the digestive fluids of multiple carnivorous plants across different lineages, including *Di. muscipula*, *Dr*. *adelae*, *N. ventrata*, and *Pinguicula × Tina* (Schulze *et al*., 2012; Lee *et al*., 2016; Kocáb *et al*., 2020; Arai *et al*., 2021). All S1‐P1 nucleases found in digestive fluids, except for one of two from *Dr. adelae*, are closely related to *Arabidopsis* ENDO2 (AtENDO2: AT1G68290). We detected accelerated protein convergence in ENDO2 orthologs in *Nepenthes* and *Cephalotus* (NgENDO2: Nepgr028074 and CfENDO2: Cfol_v3_09804). Excess convergence, reflected by a high *ω*_C_ value, was also observed between the terminal branch of *CfENDO2* and the stem branch of carnivorous Caryophyllales orthologs. This suggests that amino acid‐changing substitutions in this gene are associated with two‐step evolution, the emergence of carnivory and the subsequent establishment of pitcher leaf organizations (Fig. 5a). This two‐step pattern is similar to the evolutionary scenario previously proposed for RNase T2 (Fukushima *et al*., 2017), but was not observed for the other convergent proteins described below. *NgENDO2* showed specific expression in the digestive zone and was slightly upregulated after the feeding treatment (Fig. 5a). *CfENDO2* showed high tissue‐specific expression in the upper and the lower pitcher walls, similar to other genes encoding digestive fluid proteins (Fig. 5a). *AtENDO2* is not induced by either JA or SA, but is induced by pathogen infection (Zhang *et al*., 2020), thus can be considered a defense response gene. While its optimal pH is 6.0 and 7.0 for single‐stranded RNA (ssRNA) and ssDNA cleavage, respectively (Ko *et al*., 2012), AtENDO2 shows relatively higher enzymatic activity at acidic pH compared with other genes within the same family in *A. thaliana* (Lesniewicz *et al*., 2013), suggesting suitability for the acidic environment of digestive fluid. A total of six convergent amino acid substitutions were located near the substrate‐binding pocket (Fig. 5a; Chou *et al*., 2013). The K48R substitution is predicted to enhance catalytic efficiency toward ssDNA and double‐stranded DNA (dsDNA), as a reverse substitution (corresponding to R74K in SmNuc1 (GenBank: WP_005410840.1)) at this position has been reported to reduce catalytic efficiency to *c*. 80% and 70% for ssDNA and dsDNA, respectively (Adámková *et al*., 2024). The Y59F substitution is located at the nucleotide binding site and can probably alter the pH optimum (Koval & Dohnálek, 2018). S1‐P1 nuclease DAN1 (NCBI accession: LC699681.1), an AtENDO2 homolog in a carnivorous Caryophyllales species *Dr. adelae*, is expressed specifically in the glandular tentacle and shows a pH optimum of 4.0 for both ssRNA and ssDNA (Yu *et al*., 2023), suggesting a protein adaptation to the acidic digestive fluids. DAN1 possesses all convergent amino acid substitutions found in a pair of CfENDO2 and ancestral ENDO2 sequences of Caryophyllales carnivorous plants, except for E167D (Fig. S7). In addition to these convergent sites, CfENDO2 and NgENDO2 accumulated more convergent substitutions. These additional substitutions may refine the enzymatic activity of the ENDO2 protein further for the pitfall trap.

PGIPs have been detected in the digestive fluids of *Di. muscipula* and *Dr. adelae* (Schulze *et al*., 2012; Arai *et al*., 2021), suggesting a potential role in plant carnivory. In our analysis, we identified excess convergent amino acid substitutions in PGIPs, whose transcripts were upregulated following the feeding treatment in the two pitcher plant lineages (Fig. 5b). Notably, convergent substitutions were detected between the stem branch of five tandemly duplicated *N. gracilis PGIP* genes (Nepgr031207 to Nepgr031211) and a *C. follicularis PGIP* gene (Cfol_v3_07443), but not in the branch pair involving the common ancestor of carnivorous Caryophyllales. This pattern suggests a pitcher trap‐specific adaptation rather than the initial establishment of carnivory. *Nepenthes PGIP* genes exhibited digestive zone‐specific upregulation, whereas a *Cephalotus PGIP* gene was upregulated across all pitcher tissues except the petiole. Among the detected convergent substitutions, Q/F126M (corresponding to Y105 in *Phaseolus vulgaris* PGIP2) was located at the interaction site with fungal polygalacturonase (Xiao *et al*., 2024), potentially altering target specificity or binding affinity, or both. The remaining two convergent sites were positioned on the opposite side of the interaction interface, suggesting that they may influence other protein properties. Antifungal proteins are thought to prevent fungal growth in the digestive fluids, thereby reducing competition for nutrients (Buch *et al*., 2013). Thus, these amino acid substitutions, found exclusively in pitcher plants rather than across the entire carnivorous clade, may optimize PGIP function for the unique digestive environment of the pitfall trap.

HAK5, a potassium/proton symporter, has been previously reported in *Di. muscipula*, where its gene expression is induced by coronatine application and facilitates the absorption of prey‐derived potassium into gland cells (Scherzer *et al*., 2015). In our analysis, we identified convergent amino acid substitutions between a *N. gracilis* HAK5 ortholog (Nepgr025854) specifically expressed in the digestive zone and a *C. follicularis* HAK5 ortholog (Cfol_v3_31376) specifically expressed in the lower pitcher wall, even though there are a total of five lineage‐specific duplicates in the same clade (Fig. 5c). Nepgr025854 showed no significant response to the feeding treatment, whereas Cfol_v3_31376 was downregulated after the feeding treatment (Fig. 5c). Two detected convergent sites (F/Q188L and S407A) were in helices in the transmembrane part, and one convergent site (L/M551V) was found in the cytosolic C‐terminal domain. These substitution sites were not located at previously identified key residues essential for potassium binding in the transmembrane part (Tascón *et al*., 2020; Maierhofer *et al*., 2024) nor overlap with previously characterized auto‐inhibitory or activation domains at the C‐terminus (Ródenas *et al*., 2021), rendering them attractive candidate sites for future biochemical characterization.

Our analysis newly identified additional cases of convergent amino acid substitutions in independently evolved pitcher plants, expanding upon the previously recognized examples of digestive fluid proteins. Notably, the newly identified proteins include both secreted and nonsecreted proteins, suggesting that selective pressures extend beyond extracellular proteins to also influence membrane‐anchored and intracellular proteins that likely function within gland cells. Both *Nepenthes* gland cells and *Cephalotus* small glands possess permeable cuticles (Adlassnig *et al*., 2012), implying that gland cells are susceptible to external disturbances, which may have driven the adaptive evolution of nonsecreted proteins responsible for maintaining cellular integrity, regulating ion transport, or protecting against damage from autodigestion.

### Conclusion

Our results reveal two contrasting strategies underlying convergently evolved pitcher leaves. *Nepenthes* shows a highly compartmentalized feeding response with transcriptional upregulation of digestive fluid proteins, whereas *Cephalotus* exhibits a holistic response across tissues with transcriptional downregulation of digestive fluid proteins. These differences highlight distinct molecular routes to achieving carnivory.

While both *C. follicularis* and *N. gracilis* upregulate protein synthesis in response to prey capture, their transcriptional responses are spatially distinct. More compartmentalized responses in digestion‐related tissues in *Nepenthes* suggest that pitcher tissues are more extensively differentiated in *Nepenthes* than in *Cephalotus*. Supporting this, a greater proportion of prey‐derived nitrogen is found outside of the pitcher tissues in *N. mirabilis* (61.5%) compared with *C. follicularis* (26.1%; Schulze *et al*., 1997). This conversely indicates that the *C. follicularis* pitchers exhibit less specialization for carnivory despite their extensive morphological adaptation. Higher photosynthetic activity of *C. follicularis* pitchers than *Nepenthes* pitchers corroborates this idea (Pavlovič, 2011), yet upon prey capture, *C. follicularis* shifts the gene regulation toward carnivory by decreasing the expression of the photosynthetic‐related genes (Fig. 1e). It is intriguing to investigate the tissue‐wise feeding response in Sarraceniaceae because they rely more on prey‐derived nitrogen (76.4% in *Darlingtonia*) but have a high photosynthetic activity comparable with *C. follicularis* in pitcher leaves (Pavlovič *et al*., 2007; Karagatzides & Ellison, 2009).

The degree of specialization toward carnivory in the pitcher leaves of *N. gracilis* and *C. follicularis* is likely shaped by their distinct habitats, which differ markedly in climate and resource availability. *N. gracilis* thrives in warm, humid tropical regions where consistently high temperatures support abundant insect activity year‐round (Anu *et al*., 2009; Schultheiss *et al*., 2022; Van Dijk *et al*., 2024). In such environments, strong selective pressures likely drive the evolution of highly specialized pitcher leaves. By contrast, *C. follicularis*, native to Albany, Australia, inhabits a region with greater seasonal temperature variation (Fukushima *et al*., 2021). This species displays developmental plasticity that allows it to produce pitcher or flat leaves in response to environmental cues, indicating a flexible reliance on carnivory. In its temperate habitat, where insect availability fluctuates depending on the time of the season, the benefits of strict specialization may not be substantial. Although *Nepenthes* exhibits similar flexibility by conditionally suppressing pitcher maturation to reduce reliance on carnivory, habitat‐driven differences in prey availability offer one plausible explanation for the divergent degrees of specialization between the two pitcher plant lineages.

## Competing interests

None declared.

## Author contributions

TW and KF conceptualized the study and wrote the manuscript. KF provided plant materials and provided the in‐house analysis pipeline. TW conducted the experiments and analyzed the data.

## Disclaimer

The New Phytologist Foundation remains neutral with regard to jurisdictional claims in maps and in any institutional affiliations.

## Supporting information


**Fig. S1** The Jaccard similarity coefficients (JC) between orthogroup sets from pairs of SOM clusters related to Fig. 3.
**Fig. S2** A *PR1*‐like gene is specifically expressed in the upper and lower pitcher walls of *Cephalotus follicularis*.
**Fig. S3** Papain‐like cysteine proteases expression in the *Cephalotus follicularis* and *Nepenthes gracilis* pitcher.
**Fig. S4** Expression profiles of aspartic proteases in the *Cephalotus follicularis* and *Nepenthes gracilis* pitcher.
**Fig. S5** Transcriptional responses of jasmonic acid (JA)‐related genes to the feeding treatment in *Cephalotus follicularis* and *Nepenthes gracilis* pitchers.
**Fig. S6** Transcriptional responses of nitrogen assimilation genes to the feeding treatment in *Cephalotus follicularis* and *Nepenthes gracilis* pitchers.
**Fig. S7** Convergent sites in ENDO2 protein, including *Drosera adelae* DAN1.
**Notes S1** Potential role of immune elicitors in the constitutive expression of digestive fluid protein genes in *Cephalotus*.
**Notes S2** Feeding responses of jasmonic acid‐related genes.
**Notes S3** Upregulation of protein synthesis may reflect nitrogen assimilation.

**Table S1** Genome information sources for the 15 species used in this study.

**Table S2** Results of differential gene expression analysis across pitcher tissues in *Cephalotus follicularis*.

**Table S3** GO enrichment analysis results of DEGs in each set association in the upset plot.

**Table S4** Results of differential gene expression analysis across pitcher tissues in *Nepenthes gracilis*.

**Table S5** Orthologous gene group classification by OrthoFinder.

**Table S6** GO enrichment analysis results of DEGs in commonly upregulated or downregulated orthogroups in the lower pitcher wall of *Cephalotus follicularis* pitcher and the digestive zone of *Nepenthes gracilis* pitcher.

**Table S7** SOM clustering results based on the merged gene expression dataset including both before and after the feeding treatment.

**Table S8** GO enrichment analysis results of the SOM clusters in Table S7.

**Table S9** CSUBST analysis results with *ω*_C_ > 3 and *O*_C_^N^ > 3.

**Table S10** DDBJ entry information.
Please note: Wiley is not responsible for the content or functionality of any Supporting Information supplied by the authors. Any queries (other than missing material) should be directed to the *New Phytologist* Central Office.

## Data Availability

RNA‐seq reads are available at the DNA Data Bank of Japan (DDBJ) under BioProject no.: PRJDB15743 (Table S10). Other data and scripts are available in the article, its Supporting Information, or on Figshare (doi: 10.6084/m9.figshare.29410970).
