# Supplementary material for: Transcriptomic prey‐capture responses in convergently evolved carnivorous pitcher plants
Source: New Phytol. 2025 Dec 17;249(5):2559–73. doi: 10.1111/nph.70848 (PMC12873510; doi:10.1111/nph.70848)
Supplement: Supplementary file 1 — Fig. S1 The Jaccard similarity coefficients (JC) between orthogroup sets from pairs of SOM clusters related to Fig. 3. Fig. S2 A PR1‐like gene is specifically expressed in the upper and lower pitcher walls of Cephalotus follicularis. Fig. S3 Papain‐like cysteine proteases expression in the Cephalotus follicularis and Nepenthes gracilis pitcher. Fig. S4 Expression profiles of aspartic proteases in the Cephalotus follicularis and Nepenthes gracilis pitcher. Fig. S5 Transcriptional responses of jasmonic acid (JA)‐related genes to the feeding treatment in Cephalotus follicularis and Nepenthes gracilis pitchers. Fig. S6 Transcriptional responses of nitrogen assimilation genes to the feeding treatment in Cephalotus follicularis and Nepenthes gracilis pitchers. Fig. S7 Convergent sites in ENDO2 protein, including Drosera adelae DAN1. Notes S1 Potential role of immune elicitors in the constitutive expression of digestive fluid protein genes in Cephalotus. Notes S2 Feeding responses of jasmonic acid‐related genes. Notes S3 Upregulation of protein synthesis may reflect nitrogen assimilation. [file NPH-249-2559-s003.pdf]

***New Phytologist* Supporting Information**

Article title: Transcriptomic prey-capture responses in convergently evolved carnivorous pitcher
Authors: Takanori Wakatake and Kenji Fukushima
Article acceptance date: 22 November 2025

The following Supporting Information is available for this article:

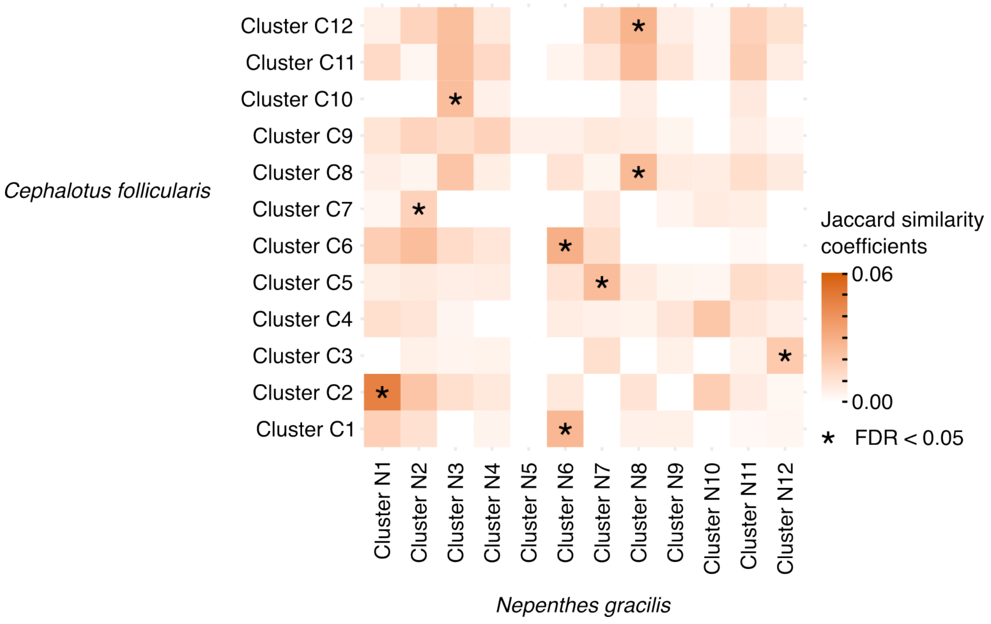

Fig. S1 The Jaccard similarity coefficients (JC) between orthogroup sets from pairs of SOM clusters related to Fig. 3. Cluster IDs are prefixed with N for *Nepenthes* and C for *Cephalotus*. Asterisks denote significantly high JC values (FDR < 0.05). *P* values were computed using permutation tests, and false discovery rates (FDRs) were adjusted using the Benjamini–Hochberg method for multiple testing correction.

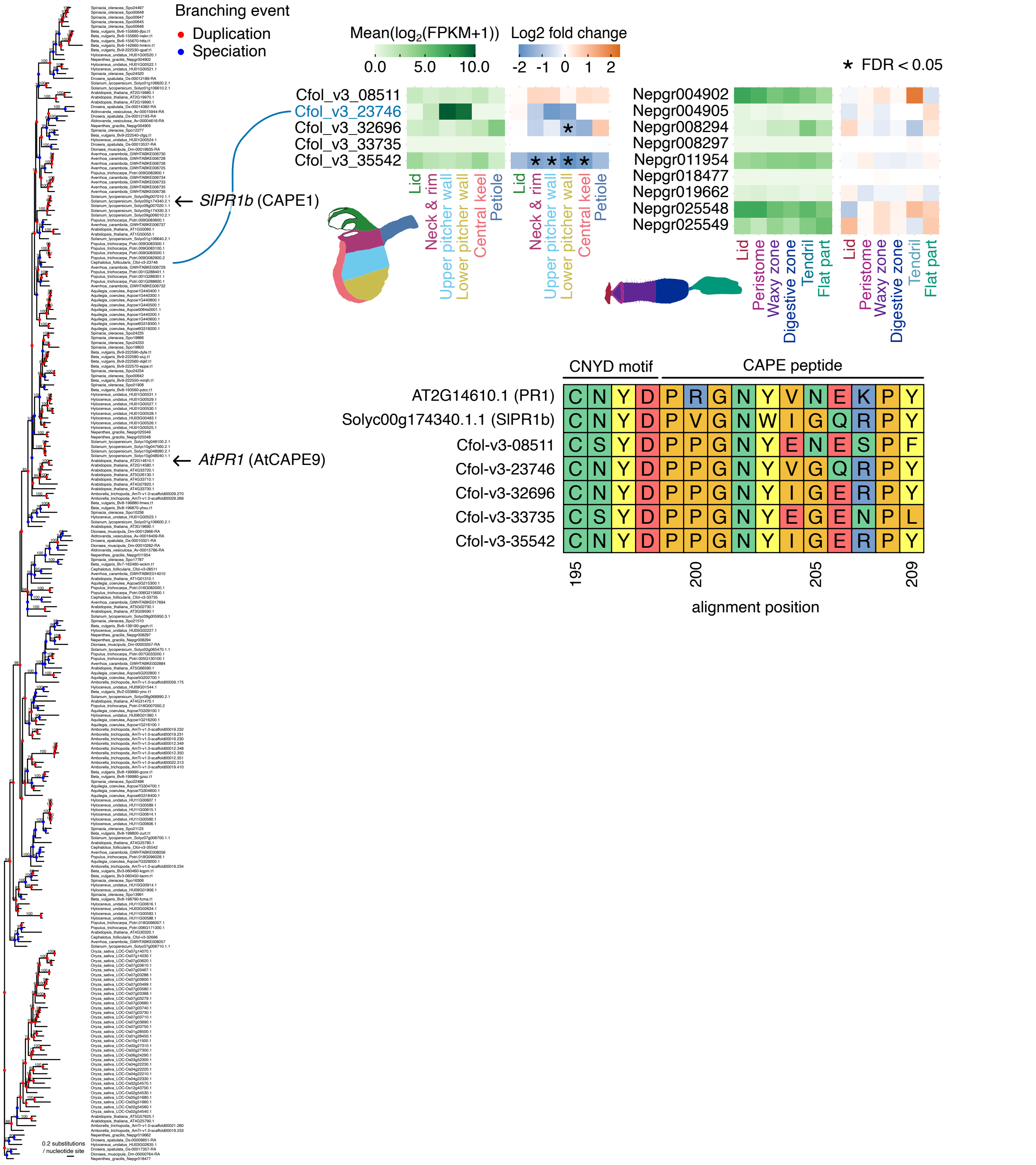

Fig. S2 A *PR1*-like gene is specifically expressed in the upper and lower pitcher walls of *C. follicularis*. The phylogenetic tree highlights the positions of previously described *PR1* genes known to be precursors of CAPE peptides (indicated by arrows). Ultrafast bootstrap values from IQ-TREE are indicated above the branches. Branches reconciled by GeneRax are marked

with a hyphen (–). Node colors represent inferred evolutionary events: speciation (blue) and gene duplication (red). Heatmaps display FPKM values under control conditions and fold changes following feeding treatment in the pitchers of *C. follicularis* and *N. gracilis*. A gene exhibiting expression patterns similar to digestive fluid proteins (corresponding to SOM cluster 5 in Fig. 3) is marked in blue. Asterisks denote significant differential expression (FDR < 0.05). The amino acid alignment illustrates the conservation of the “CNYD” motif and CAPE peptide sequences at the C-terminus of PR1 proteins. Amino acids are color-coded according to their side-chain chemical properties.

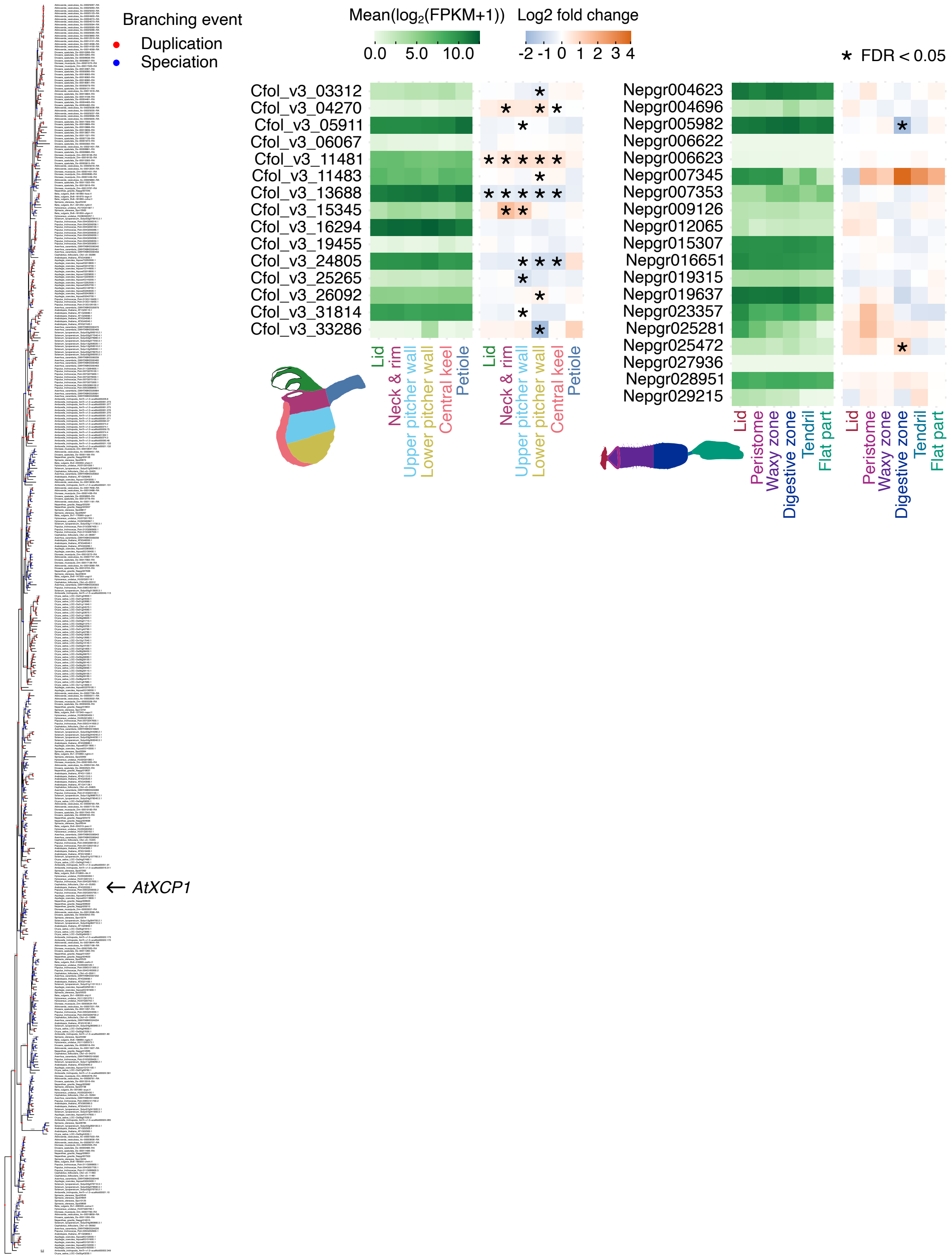

Fig. S3 Papain-like cysteine proteases expression in the *C. follicularis* and *N. gracilis* pitcher.

The phylogenetic tree highlights the position of *AtXCP1* known to produce CAPE peptides (indicated by an arrow). Ultrafast bootstrap values from IQ-TREE are indicated above the

branches. Branches reconciled by GeneRax are marked with a hyphen (–). Node colors represent inferred evolutionary events: speciation (blue) and gene duplication (red). Heatmaps display FPKM values under control conditions and fold changes following feeding treatment in the pitchers of *C. follicularis* and *N. gracilis*. Asterisks denote significant differential expression (FDR < 0.05).

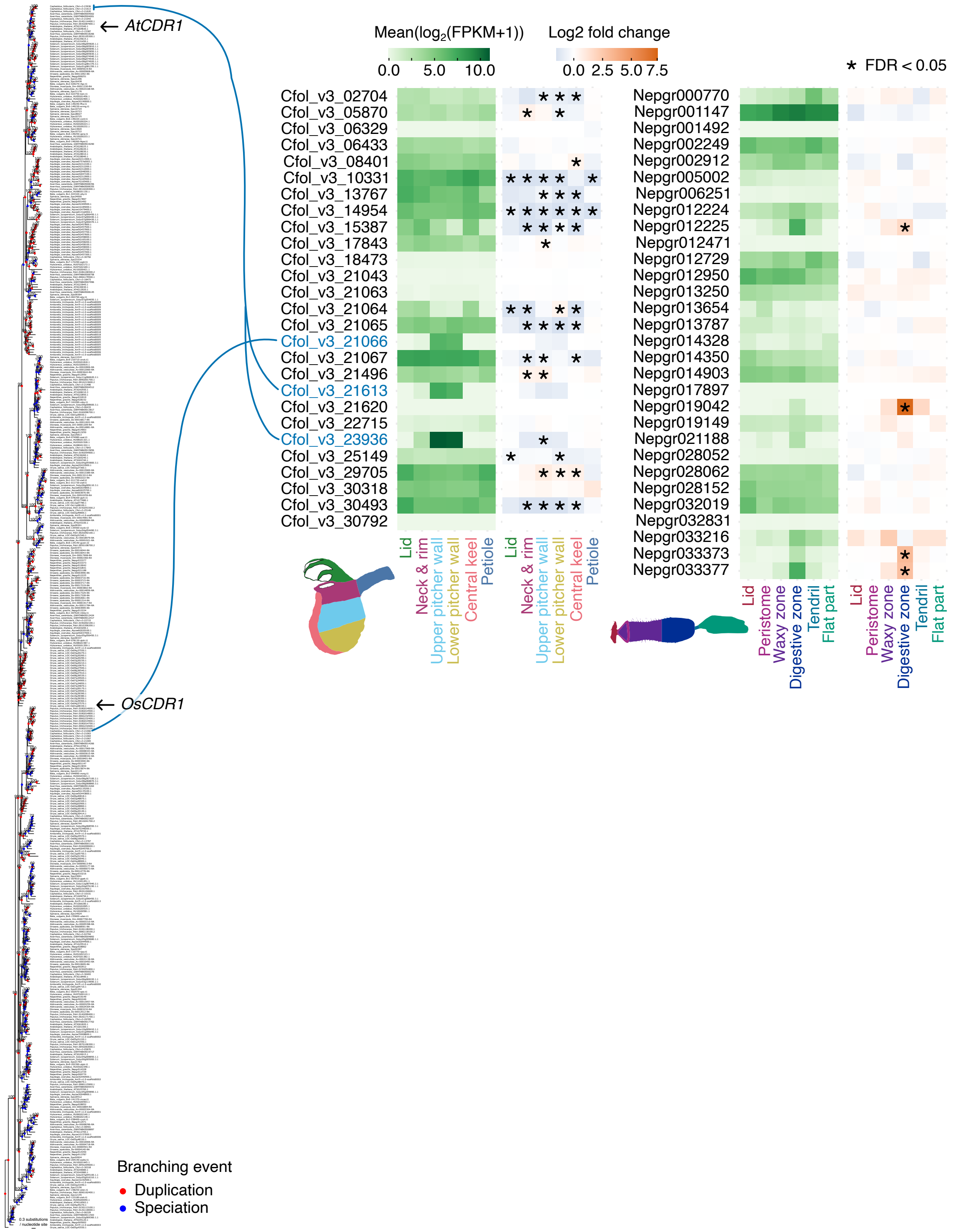

Fig. S4 Expression profiles of aspartic proteases in the *C. follicularis* and *N. gracilis* pitcher. The phylogenetic tree highlights the positions of *CDR1* genes known to enhance immune responses (indicated by arrows). Ultrafast bootstrap values from IQ-TREE are indicated above

the branches. Branches reconciled by GeneRax are marked with a hyphen (–). Node colors represent inferred evolutionary events: speciation (blue) and gene duplication (red). Heatmaps show FPKM values under control conditions and fold changes after feeding treatment in the pitchers of *C. follicularis* and *N. gracilis*. Genes exhibiting expression patterns similar to digestive fluid proteins (corresponding to SOM cluster 5 in Fig. 3) are marked in blue. Asterisks denote significant differential expression (FDR < 0.05).

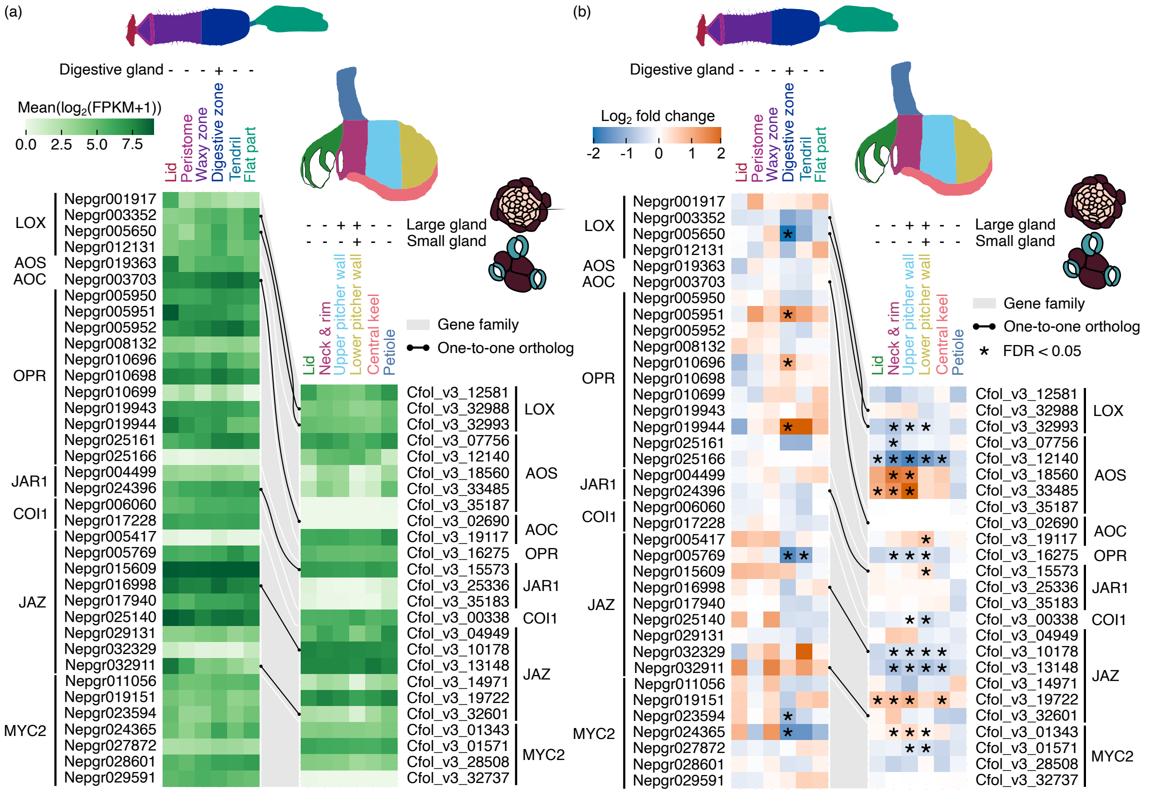

Fig. S5 Transcriptional responses of jasmonic acid (JA)-related genes to the feeding treatment in *C. follicularis* and *N. gracilis* pitchers. (a) Expression level (log_2(FPKM+1)) of the JA-related genes. (b) Log_2-fold change of the JA-related genes upon the feeding treatment. Grey ribbons connect genes in the same gene family. The black dots and lines connect the orthologous genes. Asterisks in (b) indicate FDR < 0.05 in differential gene expression analysis.

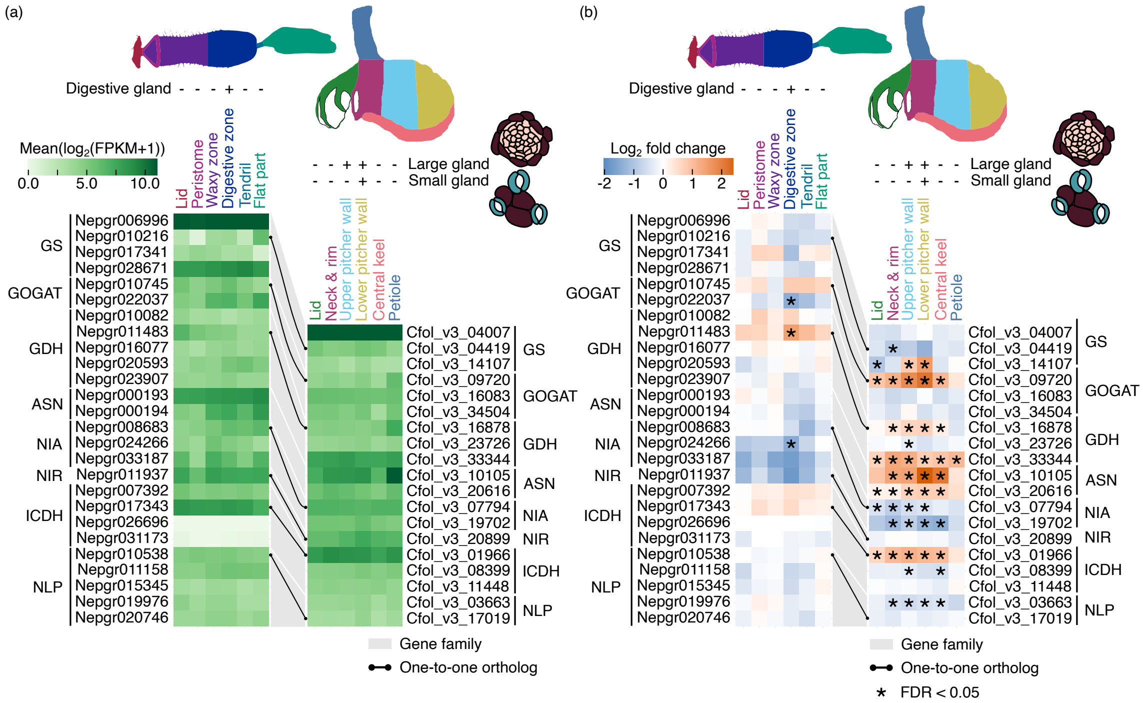

Fig. S6 Transcriptional responses of nitrogen assimilation genes to the feeding treatment in *C. follicularis* and *N. gracilis* pitchers. (a) Expression level ($\log_2$(FPKM+1)) of the nitrogen assimilation genes. (b) Log_2-fold change of the nitrogen assimilation genes upon the feeding treatment. Grey ribbons connect genes in the same gene family. The black dots and lines connect the orthologous genes. Asterisks in (b) indicate FDR < 0.05 in differential gene expression analysis.

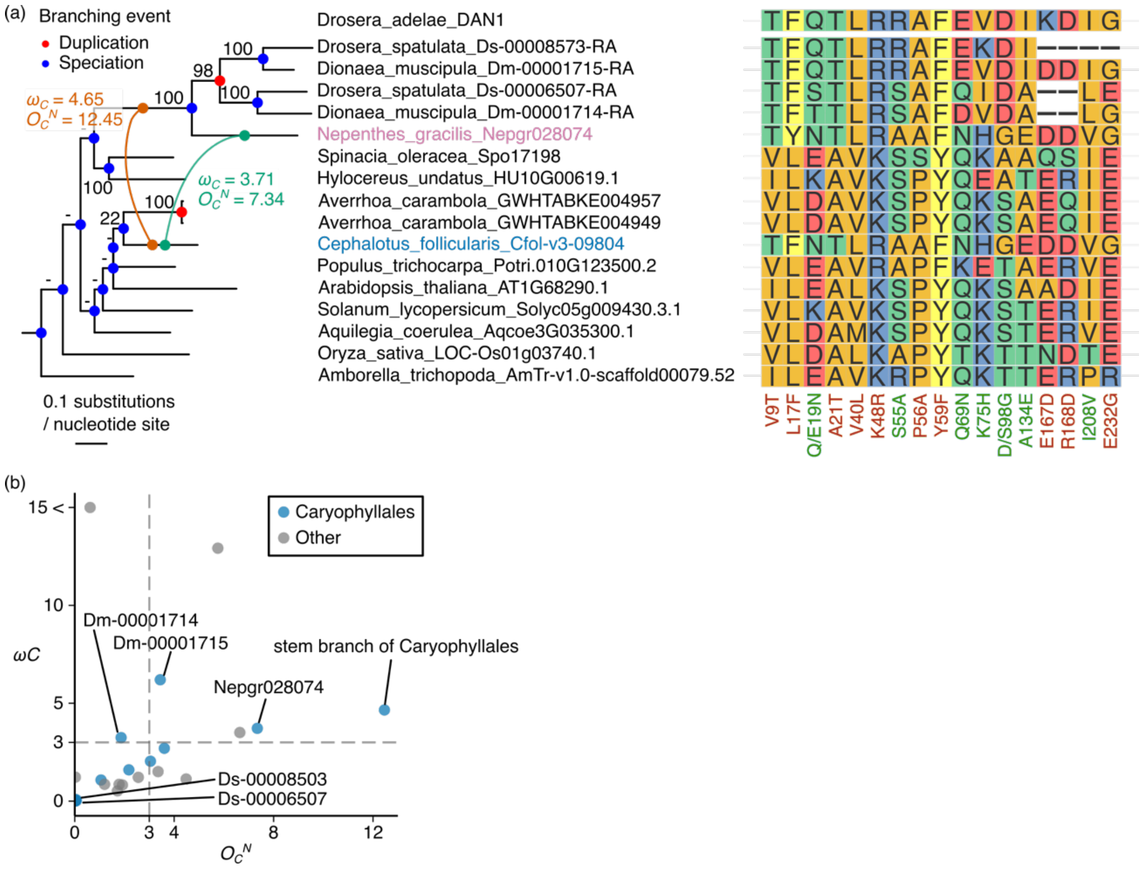

Fig. S7 Convergent sites in ENDO2 protein, including *Drosera adelae* DAN1. (a) *Cephalotus* and *Nepenthes* genes are highlighted in blue and purple, respectively. A green line connects the focal branch pair that exhibits a high convergence rate, while a brown line links the focal *Cephalotus* branch to the stem branch of carnivorous Caryophyllales ortholog. The corresponding ω_C and O_C^N (protein convergence rate and the number of amino acid changing convergent substitutions, respectively) values are indicated in matching colors. Ultrafast bootstrap values from IQ-TREE are indicated above the branches. Branches reconciled by GeneRax are marked with a hyphen (–). Node colors represent inferred evolutionary events: speciation (blue) and gene duplication (red). Amino acids are depicted with colors corresponding to their side-chain chemistry. The x-axis label indicates substitution sites. The site numbers align with those found in the PDB entry (3W52), and font colors correspond to the branch pair in the phylogenetic tree. (b) ω_C and O_C^N values of branch pairs between *CfENDO2*

(Cfol-v3-09804) and all other branches in the ENDO2 clade of OG0000836. Two dotted lines indicate thresholds ($\omega_C > 3$ and $O_C^N > 3$) used for the initial screening.

Table S1 Genome information sources for the 15 species used in this study.

Table S2 Results of differential gene expression analysis across pitcher tissues in *C. follicularis*. The merged output of DEG analyses for all sampled tissues. Tissue identity is indicated by the suffix in column names, using the following abbreviations: LID, lid; NEC, neck and rim; UPP, upper pitcher wall; LWP, lower pitcher wall; KEL, central keel; PET, petiole. gene_id: Gene identifier. m.value: Log2 fold change between the two conditions. q.value: Adjusted p-value after multiple testing correction using the Benjamini-Hochberg false discovery rate. estimatedDEG: Binary indicator of differential expression status (1 = DEG, 0 = non-DEG).

Table S3 GO enrichment analysis results of DEGs in each set association in the upset plot. Only GO terms with more than three associated genes and an adjusted q-value < 0.05 (Benjamini-Hochberg method) are included. Sheet names indicate gene set associations corresponding to those shown in Fig. 1. Tissue name abbreviations follow the same format as described in Supporting Information Table S1

Table S4 Results of differential gene expression analysis across pitcher tissues in *N. gracilis*. The merged output of DEG analyses for all sampled tissues. Tissue identity is indicated by the suffix in column names, using the following abbreviations: LID, lid; PRS, peristome; WXZ, waxy zone; DGZ, digestive zone; TDR, tendril; FLP, flat part.

Table S5 Orthologous gene group classification by OrthoFinder. The column labeled “HOG” indicates

the hierarchical orthogroup (HOG) assignments determined at the node representing the most recent common ancestor of *C. follicularis* and *N. gracilis*.

Table S6 GO enrichment analysis results of DEGs in commonly upregulated or downregulated orthogroups in the lower pitcher wall of *C. follicularis* pitcher and the digestive zone of *N. gracilis* pitcher. Only GO terms with more than three associated genes and an adjusted q-value < 0.05 (Benjamini-Hochberg method) are included. Sheet name includes abbreviations as follows: Cf, *Cephalotus follicularis*; Ng, *Nepenthes gracilis*. Tissue name abbreviations follow the same format as described in Supporting Information Table S1 and S3.

Table S7 SOM clustering results based on the merged gene expression dataset including both before and after the feeding treatment. Each gene is assigned to a cluster, indicated in the "unit.class" column. Mean expression values for each tissue are provided. "Zmean" columns represent Z-scores calculated from the mean expression values across tissues. Tissue names follow the abbreviations used in Supporting Information Table S1 and S3. Treatments are abbreviated as follows: CON, control; WOR, mealworm extract.

Table S8 GO enrichment analysis results of the SOM clusters in supplementary table S6**.** Only GO terms with more than three associated genes and an adjusted q-value < 0.05 (Benjamini-Hochberg method) are included. Sheet name includes abbreviations as follows: Cf, *Cephalotus follicularis*; Ng, *Nepenthes gracilis*.

Table S9 CSUBST analysis results with $\omega_C > 3$ and $O_C^N > 3$. Each row represents a result of one branch pair. The numerical suffix (e.g., _1 or _2) indicates whether the column pertains to branch_id_1 or

branch_id_2. branch_id_1, branch_id_2: Identifiers for the focal branches being analyzed. omegaCany2spe: The rate of combinatorial codon substitutions from any ancestral codons to specific descendant codons (equivalent to ω_C in the manuscript). OCNany2spe: The number of combinatorial substitutions (corresponds to O_C^N in the manuscript). spnode_coverage: species coverage on the branch sprot_recname: functional annotation from RPS-BLAST.

Table S10 DDBJ entry information.

Notes S1 Potential role of immune elicitors in the constitutive expression of digestive fluid protein genes in *Cephalotus*. *Cephalotus* plants grown axenically, and therefore free of biotic stimuli, still show constitutive expression of digestive-fluid protein genes. Many of these genes are evolutionarily derived from defense-related loci (Fukushima et al. 2017), so the mechanism sustaining their expression remains unclear. One possible explanation is the continuous production of immune elicitors, such as *PATHOGENESIS-RELATED PROTEIN 1* (*PR1*), by apoplastic proteases including *CONSTITUTIVE DISEASE RESISTANCE* (*CDR1*). For example, in *Solanum lycopersicum*, CAPE1 peptide derived from a PR1 protein called SlPR1b induces the expression of defense response genes, including members of PR protein families (Chen et al. 2014). In *A. thaliana*, *XYLEM CYSTEINE PEPTIDASE 1* (*XCP1*) is recognized as a main protease responsible for producing AtCAPE9 peptide from PR1 proprotein (Chen et al. 2023). Notably, one *Cephalotus PR1* gene was preferentially expressed in the upper and lower pitcher walls, mirroring the expression pattern of genes encoding digestive fluid proteins (Supporting Information Fig. S2). This gene harbors the “CNYD” substrate motif targeted by cysteine peptidase at its C-terminal. Although we did not observe clear tissue specificity, multiple cysteine peptidases were expressed highly across pitcher tissues (Supporting Information Fig. S3). This suggests the constant production of the CAPE peptide from PR1 protein via protease activity, potentially eliciting a sustained immune response in the pitchers. Similarly, aspartic protease CDR1 is known to induce defense

responses. In *A. thaliana*, *CDR1-D* mutant, which overexpresses *CDR1*, exhibits enhanced expression of *PR* genes (Xia et al. 2004). Its substrate is still unknown, but its functional conservation in rice has been reported (Prasad et al. 2009). Two *CDR1*-like genes in *C. follicularis* showed similar expression pattern to genes encoding digestive fluid proteins (Supporting Information Fig. S4), suggesting a role in producing immune elicitors potentially linked to digestive fluid proteins.

Notes S2 Feeding responses of jasmonic acid-related genes. In the carnivorous Caryophyllales lineage, including *Nepenthes*, jasmonic acid (JA) signaling mediates feeding responses (Nakamura et al. 2013; Mithöfer et al. 2014; Buch et al. 2015; Böhm et al. 2016; Yilamujiang et al. 2016; Krausko et al. 2017). However, in *C. follicularis*, a member of the Oxalidales order, the endogenous JA level does not substantially change in response to prey capture, and coronatine, a bacterial toxin that mimics JA, does not induce digestive enzyme accumulation (Pavlovič et al. 2024). To further investigate the potential involvement of the JA signaling pathway in prey capture response in *C. follicularis*, we surveyed the transcriptional responses of genes involved in the JA biosynthesis and signaling (Wasternack and Song 2017; Wasternack and Feussner 2018) upon the feeding treatment. A few genes involved in JA biosynthesis, including those encoding allene oxide synthase (AOS), allene oxide cyclase (AOC), and jasmonate–amido synthetase (JAR1), were upregulated after the feeding treatment, but their induction occurred in different tissues (Supporting Information Fig. S5). It is unlikely that JA biosynthesis was activated in response to the feeding treatment. In addition, *CORONATINE-INSENSITIVE 1* (*COI1*) and *JASMONATE-ZIM DOMAIN* (*JAZ*), encoding co-receptor complex of JA (Chini et al. 2007; Thines et al. 2007; Fonseca et al. 2009), were also not significantly induced except for one *JAZ* gene (Cfol_v3_19722), which showed only weak induction. In *N. gracilis*, three distinct genes encoding 12-oxophytodienoic acid (OPDA) reductase 3 (OPR3), required for JA biosynthesis, were specifically upregulated in the digestive zone. In contrast, other JA biosynthesis genes were not detected as DEGs across any tissues except for one gene encoding 13-lipoxygenase (LOX) which was instead downregulated (Supporting

Information Fig. S5). Signaling components were slightly upregulated in the lid and the waxy zone, but those changes were not significant.

Notes S3 Upregulation of protein synthesis may reflect nitrogen assimilation. Upregulation of genes involved in protein synthesis is a major feeding response shared between the two pitcher plant lineages (Supporting Information Table S6). In *N. gracilis*, this response is thought to be associated with the rapid production of digestive enzymes triggered by the feeding treatment (Saul et al. 2023). However, this scenario is not likely in *C. follicularis* because its digestive fluid proteins were rather downregulated after the feeding treatment (Fig. 4c). Alternatively, we propose that the upregulation of protein synthesis in *C. follicularis* may be linked to the process of nitrogen assimilation. In *A. thaliana*, nitrogen assimilation- and protein synthesis-related genes are quickly upregulated under nitrogen-replete conditions (Scheible et al. 2004). Similarly in *C. follicularis*, genes associated with the glutamine synthetase-glutamate synthase pathway, which is the first step of nitrogen assimilation (Masclaux-Daubresse et al. 2010), were upregulated particularly in the lower pitcher wall (Supporting Information Fig. S6), potentially driven by prey-derived proteins or their degradation products such as ammonium.

References

Chen Y-L, Lee C-Y, Cheng K-T, Chang W-H, Huang R-N, Nam HG, Chen Y-R. 2014. Quantitative Peptidomics Study Reveals That a Wound-Induced Peptide from PR-1 Regulates Immune Signaling in Tomato. Plant Cell 26:4135–4148.

Chen Y-L, Lin F-W, Cheng K-T, Chang C-H, Hung S-C, Efferth T, Chen Y-R. 2023. XCP1 cleaves Pathogenesis-related protein 1 into CAPE9 for systemic immunity in Arabidopsis. Nat. Commun. 14:4697.

Chini A, Fonseca S, Fernández G, Adie B, Chico JM, Lorenzo O, García-Casado G, López-Vidriero I, Lozano FM, Ponce MR, et al. 2007. The JAZ family of repressors is the missing link in jasmonate signalling. *Nature* 448:666–671.

Fonseca S, Chini A, Hamberg M, Adie B, Porzel A, Kramell R, Miersch O, Wasternack C, Solano R. 2009. (+)-7-iso-Jasmonoyl-L-isoleucine is the endogenous bioactive jasmonate. *Nat. Chem. Biol.* 5:344–350.

Krausko M, Perutka Z, Šebela M, Šamajová O, Šamaj J, Novák O, Pavlovič A. 2017. The role of electrical and jasmonate signalling in the recognition of captured prey in the carnivorous sundew plant *Drosera capensis*. *New Phytol.* 213:1818–1835.

Masclaux-Daubresse C, Daniel-Vedele F, Dechorgnat J, Chardon F, Gaufichon L, Suzuki A. 2010. Nitrogen uptake, assimilation and remobilization in plants: challenges for sustainable and productive agriculture. *Ann. Bot.* 105:1141–1157.

Mithöfer A, Reichelt M, Nakamura Y. 2014. Wound and insect-induced jasmonate accumulation in carnivorous *Drosera capensis*: two sides of the same coin. *Plant Biol.* 16:982–987.

Nakamura Y, Reichelt M, Mayer VE, Mithöfer A. 2013. Jasmonates trigger prey-induced formation of 'outer stomach' in carnivorous sundew plants. *Proc. R. Soc. B Biol. Sci.* 280:20130228.

Prasad BD, Creissen G, Lamb C, Chattoo BB. 2009. Overexpression of Rice (Oryza sativa L.) *OsCDR1* Leads to Constitutive Activation of Defense Responses in Rice and Arabidopsis. Mol. Plant-Microbe Interact. 22:1635–1644.

Scheible W-R, Morcuende R, Czechowski T, Fritz C, Osuna D, Palacios-Rojas N, Schindelasch D, Thimm O, Udvardi MK, Stitt M. 2004. Genome-Wide Reprogramming of Primary and Secondary Metabolism, Protein Synthesis, Cellular Growth Processes, and the Regulatory Infrastructure of Arabidopsis in Response to Nitrogen. *Plant Physiol.* 136:2483–2499.

Thines B, Katsir L, Melotto M, Niu Y, Mandaokar A, Liu G, Nomura K, He SY, Howe GA, Browse J. 2007. JAZ repressor proteins are targets of the SCF^{COI1} complex during jasmonate signalling. *Nature* 448:661–665.

Wasternack C, Feussner I. 2018. The Oxylipin Pathways: Biochemistry and Function. *Annu. Rev. Plant Biol.* 69:363–386.

Wasternack C, Song S. 2017. Jasmonates: biosynthesis, metabolism, and signaling by proteins activating and repressing transcription. *J. Exp. Bot.* 68:1303–1321.

Xia Y, Suzuki H, Borevitz J, Blount J, Guo Z, Patel K, Dixon RA, Lamb C. 2004. An extracellular aspartic protease functions in Arabidopsis disease resistance signaling. EMBO J. 23:980–988.
